# SVFX: a machine learning framework to quantify the pathogenicity of structural variants

**DOI:** 10.1186/s13059-020-02178-x

**Published:** 2020-11-09

**Authors:** Sushant Kumar, Arif Harmanci, Jagath Vytheeswaran, Mark B. Gerstein

**Affiliations:** 1 Program in Computational Biology and Bioinformatics, Yale University, New Haven, CT 06520 USA; 2 Department of Molecular Biophysics and Biochemistry, Yale University, New Haven, CT 06520 USA; 3 Center for Precision Health, School of Biomedical Informatics, University of Texas Health Science Center, Houston, TX 77030 USA; 4 Department of Computing and Mathematical Sciences, California Institute of Technology, Pasadena, CA 91125 USA; 5 Department of Computer Science, Yale University, 260/266 Whitney Avenue, PO Box 208114, New Haven, CT 06520 USA

## Abstract

There is a lack of approaches for identifying pathogenic genomic structural variants (SVs) although they play a crucial role in many diseases. We present a mechanism-agnostic machine learning-based workflow, called SVFX, to assign pathogenicity scores to somatic and germline SVs. In particular, we generate somatic and germline training models, which include genomic, epigenomic, and conservation-based features, for SV call sets in diseased and healthy individuals. We then apply SVFX to SVs in cancer and other diseases; SVFX achieves high accuracy in identifying pathogenic SVs. Predicted pathogenic SVs in cancer cohorts are enriched among known cancer genes and many cancer-related pathways.

**Supplementary information:**

**Supplementary information** accompanies this paper at 10.1186/s13059-020-02178-x.

## Background

Large-scale whole-genome sequencing is providing high-resolution maps of genomic variation in various disease-specific studies [1–4]. These studies have created extensive catalogs of genomic alterations that comprise single-nucleotide changes [single-nucleotide variants (SNVs) or single-nucleotide polymorphisms (SNPs)], insertions and deletions (indels, ranging between 1 and 50 bp), and structural variants (SVs, exceeding 50 bp). SVs are often classified as imbalanced or balanced based on their effect on the copy number profile. Imbalanced SVs result in copy number changes through large deletions, duplications, or insertions. In contrast, balanced SVs (such as translocations and inversions) do not alter the copy number profile of an individual. Despite their lower frequency, SVs contribute more nucleotide-level changes than the aggregated frequency of SNVs/SNPs and indels [5].

Due to their large size, SVs play a vital role in the progression of various diseases, including cancer, intellectual disabilities, and neurodegenerative diseases [4]. In the context of cancer, these rearrangements often lead to the removal or fusion of genes and their cis-regulatory elements, thereby disrupting essential functions, including cell growth, differentiation, signaling, and apoptosis [6]. Despite their important roles in various diseases, ascertaining the pathogenicity and establishing mechanistic links between SVs and disease progression remain challenging [7]. These challenges are exacerbated by difficulties associated with the accurate identification of SVs and their precise breakpoint [8].

Prior studies aiming to quantify the pathogenicity and ascertain the roles of genomic variations in disease have primarily been limited to point mutations and small indels [9–13]. In contrast, only a handful of studies have sought to evaluate the molecular consequences of SVs [14]. Initial attempts to characterize the molecular impact of SVs were limited to annotating genes that overlap with germline SVs, without assigning pathogenicity scores. A recent study [14] leveraged genome-wide per-base pathogenicity scores [9] (initially designed for measuring the impact of single-nucleotide changes) to assign impact scores for germline SVs. Despite these early efforts, there is a clear need for a systematic framework to clarify the molecular and functional consequences of SVs and their roles in human disease.

To address this challenge, we present an integrative supervised machine learning framework (SVFX) to assign pathogenic scores to somatic and germline SVs. We hypothesized that the underlying genomic and epigenomic features of pathogenic SVs are very different from those of benign SVs. Moreover, we expected that these differences could be sensitively detected only in a tissue-specific context. Thus, we built machine learning models that assign a pathogenic score by comparing the genomic and tissue-specific epigenomic features of a given SV with those of known benign SVs. Our framework is highly flexible and can be applied to identify pathogenic somatic and germline SVs in cancer as well as other diseases. Toward this end, we utilized high-quality somatic and germline SV data from the Pan-Cancer Analysis of Whole Genomes (PCAWG) Project [15], Genome Sequencing Program (GSP), ClinVar database [16], Genome Aggregation Database (gnomAD) [17], and 1000 Genomes (1KG) Project [5, 18] to train our machine learning models. Additionally, we employed tissue-specific epigenomic data from the Epigenome Roadmap [19], various genomic element annotations [20, 21], and cross-species conservation metrics [22] to build our machine learning models.

Overall, our approach achieved high accuracy in discriminating pathogenic somatic SVs from benign variants for large deletions (mean area under the curve [AUC] of 0.865) and duplications (mean AUC of 0.835) across multiple cancer types. Additionally, our germline models attained good accuracy in identifying pathogenic germline SVs in cancer, ClinVar, cardiovascular (CVD), and inflammatory bowel disease (IBD) cohorts. In particular, in cancer genomes, our somatic model identified pathogenic deletions and duplications that are enriched among key pathways and biological processes, including cell cycle regulation, cell differentiation, and signal transduction. Additionally, for somatic models in which we excluded conservation and known cancer gene annotations as features, we found that high-scoring (pathogenic) SVs tend to influence highly conserved regions of the genome and are enriched among known cancer genes. This observation provides further evidence for the robustness of our approach in identifying pathogenic SVs. Finally, we annotate and discuss examples of somatic and common disease SVs that we identified as highly pathogenic using our method.

## Results

### Training dataset and SV impact workflow

**Fig. 1:**
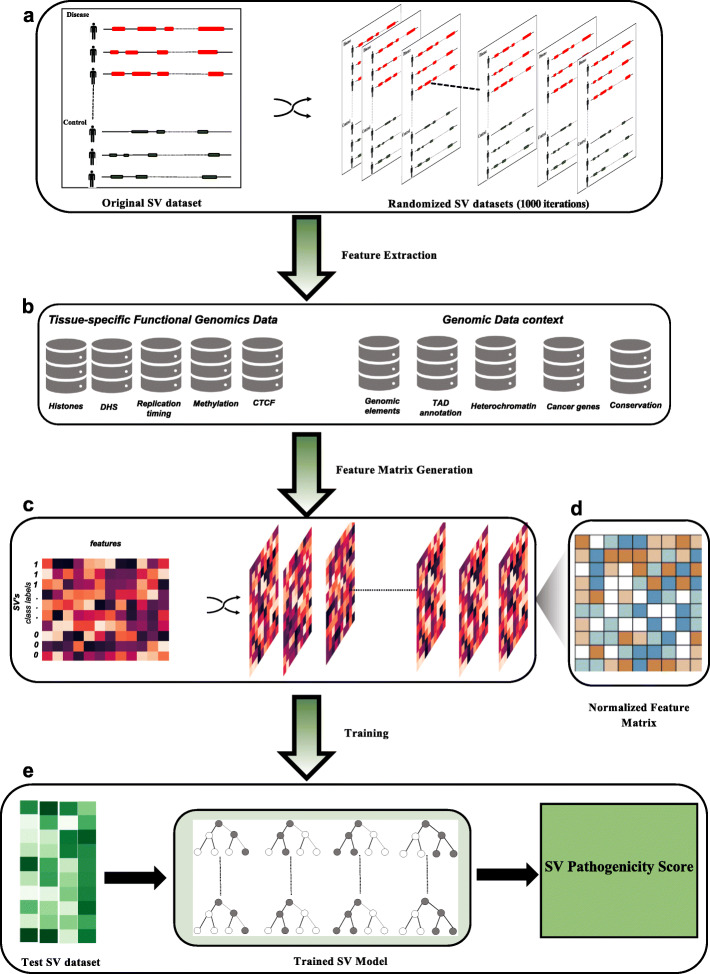
Machine learning-based workflow of SVFX to identify pathogenic SVs. The original SV dataset consists of disease/case and control SVs. In our somatic model, disease SVs correspond to somatic SVs found in a cancer cohort and control SVs correspond to SVs found in the 1KG SVs. We randomly select SVs from the 1KG SV dataset such that the number of somatic SVs and control SVs matches. Similarly, for our germline model, we have (1) disease germline SVs identified in a specific disease cohort and (2) control SVs that correspond to common SVs in the 1KG SV dataset. For both germline and somatic models, we generate 1000 random iterations of the original disease and control dataset. These permuted SVs are later utilized for generating a *Z*-score-normalized feature matrix

For each disease cohort, we built separate somatic and germline models. In the somatic SV models, the training sets consisted of cancer and control (i.e., benign SVs from the 1KG project) SVs (Fig. 1a). For the germline cancer model, we subsampled germline SVs for each cancer cohort such that the number of germline SVs in the disease set matched that of the common SVs (global allele frequency > 0.5%) in the 1KG SV dataset [5]. Additionally, the CVD cohort in our study had a unique advantage of being a careful case-control study. Thus, instead of using common 1KG SVs as benign variants, we utilized common SVs belonging to the control group from this study as the benign SV dataset. For the IBD cohort model, we used common SVs belonging to the gnomAD-SV database as the benign SVs. Finally, we utilized likely pathogenic SVs and benign SVs from the ClinVar database to train our ClinVar model.

Previous studies [23–26] have shown that the distribution of somatic and germline SVs depends on a complex interplay among different mechanistic biases, originating from underlying chromosome conformations [25], DNA accessibility [24, 27], functional embedding [5], methylation profiles [26], and cross-species conservation. For instance, disease SVs disrupt topologically associated domains (TADs) that influence the gene-enhancer interaction, leading to various diseases [25]. Similarly, the methylation status [26] and DNA accessibility [27] of genomic regions have been previously associated with the emergence of somatic and germline SVs. Furthermore, the disruption of coding and noncoding genomic elements—including promoters, untranslated regions (UTRs), and enhancers—along with the disruption of highly conserved genomic regions is likely to play a critical role in disease progression [5]. Despite these strong correlations, the exact mechanism by which a pathogenic SV drives disease progression remains elusive for many diseases. Thus, in this work, we adopted a data-driven approach in which we built agnostic machine learning models, incorporating various genomic and epigenomic features underlying SVs. Further, we hypothesized that the genomic and epigenomic profiles of pathogenic SVs are highly distinct from the profiles of benign SVs.

Accordingly, we built feature matrices for our somatic and germline models, where each row corresponded to an SV and each column to a distinct feature. These feature matrices consist of important epigenomic features, including average histone mark signals, methylation levels, CTCF signals, open chromatin marks, and replication timing data, that overlap with SVs in the disease and benign datasets (Additional file 1: Table S1). Furthermore, we integrated relevant genomic element annotations, including the fraction of overlap between SVs and the coding region, 3′ and 5′ UTRs, splice sites, and promoter regions of genes in the human genome. The feature matrices also captured additional annotations, including TAD boundary definitions, heterochromatic regions, fragile sites, sensitive sites, and ultra-conserved regions in the genome (Fig. 1b).

As a unique challenge in feature-based representation, SVs exhibited an apparent disparity in their length distributions in disease cohorts (particularly somatic cohorts) and in the 1KG dataset (Additional file 2: SI Fig. S1). These differences in length distributions for disease and benign SVs may implicitly influence various feature values. Thus, to avoid any bias in the training features, we uniformly shuffled the SVs in the disease cohorts and in the benign SV dataset to generate null distributions of feature values (Fig. 1c). We next utilized these shuffled SVs to transform each feature value in the original feature matrices (consisting of disease and benign SVs) to obtain *Z*-score-normalized feature matrices using the null distribution of each corresponding element (Fig. 1d). While *Z*-score-based normalization compensates for the length bias, we sought to ensure that our models assign high pathogenic scores to extremely long SVs, such as chromosomal arm-level variants. Thus, we appended the length of each SV as an explicit feature in our *Z*-score-normalized feature matrices.

For each disease cohort, we utilized these updated *Z*-score-normalized feature matrices to train supervised machine learning models using random forests for somatic and germline SVs separately (Fig. 1e). Finally, we validated these trained models using tenfold cross-validation and a holdout test dataset for each cohort.

### Accuracy assessment of somatic cancer models

We applied our method to quantify the pathogenic scores of somatic SVs in six different cancer cohorts, including breast, ovarian, liver, esophageal, stomach, and skin cancer. We selected these cohorts because tissue-specific epigenomic data were available and because these cancer types exhibit a significant number of SVs, which are needed for training and testing the model. Subsequently, we evaluated whether these models could distinguish pathogenic somatic SVs from benign SVs. Intuitively, one would expect our somatic model to assign high impact scores to cancer SVs and low pathogenicity scores to benign 1KG SVs. Moreover, we would expect SVs with high pathogenic scores to act as cancer drivers, whereas low-scoring SVs are likely to be passengers with little or no consequence on tumor progression. We quantitatively assessed this hypothesis using tenfold cross-validation and a holdout test dataset for each cancer cohort. Briefly, we measured the average areas under the receiver operator characteristic (auROC) and the precision-recall (auPR) curves.

**Fig. 2:**
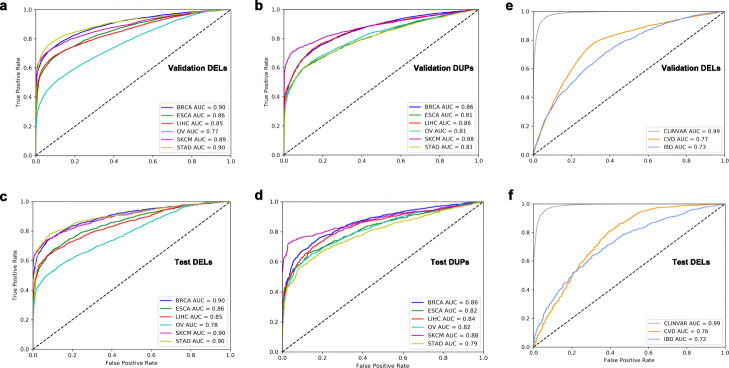
Performance evaluation for somatic models to predict pathogenic SVs in various cancer types. This figure presents area auROCs based on the validation datasets for large deletions (**a**) and duplications (**b**) in six different cancer cohorts including breast adenocarcinoma (BRCA), esophageal carcinoma (ESCA), liver (LIHC), ovary (OV), skin melanoma (SKCM), and stomach (STAD) cancers. Similarly, auROC plots are shown for test datasets associated with large deletions (**c**) and duplications (**d**) in six different cancer cohorts. Finally, auROC plots are shown for pathogenic SVs in the validation (**e**) and testing dataset (**f**) in the ClinVar, CVD, and IBD cohort datasets

Overall, our somatic models for both deletions and duplications performed very well. For somatic deletions with the tenfold cross-validation strategy, the mean auROC and auPR values across all six cancer types were 0.861 and 0.892, respectively (Fig. 2a, Additional file 2: SI Fig. S2a). Furthermore, the mean auROC and auPR values for the somatic duplication models across the six cancer cohorts were 0.835 and 0.87, respectively (Fig. 2b, Additional file 2: SI Fig. S3a). In addition to tenfold cross-validation, we assessed the performance of our somatic deletion and duplication models in a holdout test dataset. Overall, we observed comparable performance, with average auROC values of 0.865 and 0.835 across the six cancer types for deletions and duplications, respectively (Fig. 2c, d). The average auPR values for the holdout test data were also very similar to those for tenfold cross-validation. Our models achieved mean auPR values of 0.87 and 0.89 across the six cancer types for deletions and duplications, respectively (Additional file 2: SI Fig. S2b & S3b). Furthermore, we quantified the pathogenic score for large deletions and duplications that are predicted to be driver events on a pan-cancer level using a recurrence-based analysis [28]. As expected, our workflow assigned a high pathogenic score (average score greater than 0.85 across different somatic models) to each putative driver event (Additional file 1: Table S2). We note that, to a certain extent, the use of *Z*-score-normalized feature matrices helped us avoid implicit bias in our somatic models due to differences in the length distribution of cancer-associated and 1KG SVs. However, we also included the SV length as an explicit feature in our final somatic models to assign high pathogenicity scores to extremely large SVs, such as chromosomal arm deletions and duplications. The inclusion of SV length as an explicit feature can potentially bias the predictive performance of our model. Thus, we quantified the predictability differences for the original models when the SV length was removed as a feature. Overall, we found that the AUC value was 3% lower, on average, for truncated somatic models that lacked SV length as a specific feature (Additional file 2: SI Fig. 4).

Finally, we evaluated the contribution of each feature to the performance of our somatic deletion and duplication models. We observed that SV length and overlap with ultra-conserved regions were the most significant contributors to the predictive performance of the model (Additional file 2: SI Fig. S5). Additionally, the predictability of the somatic deletion models depended on other noncoding and epigenomic features, including overlap with 3′ and 5′ UTRs, sensitive regions, and H3K4me3 signals, suggesting an essential influence of SVs on cis-regulatory elements. Similarly, the predictive performance of the somatic duplication models primarily depended on an overlap with known cancer genes, heterochromatin annotation, UTRs, and sensitive regions (Additional file 2: SI Fig. S6).

### Accuracy assessment of germline cancer models

In addition to somatic models, we built germline SV models to identify pathogenic germline SVs in six cancer cohorts. We assessed the predictive accuracy of our germline models in cancer cohorts using tenfold cross-validation and a holdout test dataset. Similar to the somatic deletion models, we observed good performance for our germline deletion models in the cancer cohorts. For tenfold cross-validation, the mean auROC and auPR values across the six cancer types were 0.79 and 0.74, respectively (Additional file 2: SI Fig. S7a & S8a). Additionally, we observed similar auROC and auPR values among the different cancer types. Among our test datasets, the average auROC and auPR values across the different cancer cohorts were approximately 0.8 and 0.75, respectively (Additional file 2: SI Fig. S7b & S8b). We note that the ovarian cancer cohort primarily drove the slight improvement of the model in the test dataset, with auROC and auPR values of 0.86 and 0.84, respectively (Additional file 2: SI Fig. S7b & S8b).

Similar to the somatic models, we quantified the prediction contribution for each feature in our cancer germline deletion models. We observed that the SV length had the greatest contribution to our cancer germline predictions. Additionally, we found substantial contributions from sensitive regions, cancer gene overlap, H3K9me3, and 3′ UTR overlap features (Additional file 2: SI Fig. S9).

### Accuracy assessment of germline models for common and rare diseases

We note that our current framework is highly flexible and can be easily applied to identify pathogenic germline SVs for various common and rare diseases. Thus, we applied our approach to detect pathogenic germline SVs in CVD and IBD cohorts (included in the GSP [29]). Furthermore, we identified putative pathogenic SVs in the ClinVar SV dataset, which is enriched with rare disease-associated SVs. Overall, our germline deletion model in the CVD cohort achieved good predictive accuracy for the tenfold cross-validation approach, with mean auROC and auPR values of 0.77 and 0.74, respectively (Fig. 2e, Additional file 2: SI Fig. S10a). Similarly, our model performed very well in identifying pathogenic deletions in the testing dataset for this cohort, with mean auROC and auPR values of 0.76 and 0.84, respectively (Fig. 2f, Additional file 2: SI Fig. S10b).

Compared with the CVD cohort, the IBD cohort had fewer deletions. Furthermore, we utilized large deletions in the recently released gnomAD-SV dataset as a control for the germline IBD model. Overall, due to the lower number of deletions in our training and testing datasets, we observed a slight decrease in the performance of our IBD-specific germline model, which had mean auROC and auPR values of 0.73 and 0.71, respectively, based on the tenfold cross-validation approach (Fig. 2e, Additional file 2: SI Fig. S11). Our model’s performance on the testing dataset for this cohort was comparable, with a mean auROC and auPR value of 0.72 (Fig. 2f, Additional file 2: SI Fig. S11). Finally, we utilized known pathogenic and benign SVs, as reported in the ClinVar SV database, to build a clinvar-specific germline model. We note that ClinVar database includes SVs from a wide range of diseases. Thus, we utilized functional and epigenomics data from a generic cell line (GM12878) to train our ClinVar germline models. Compared with data for common diseases such as CVD and IBD, the ClinVar SV database is highly enriched with rare and likely deleterious SVs. Thus, our model achieved near-perfect accuracy and recall (auROC and auPR values of 0.99) while distinguishing pathogenic ClinVar deletions and duplications from benign SVs (Fig. 2e, f; Additional file 2: SI Fig. S12).

Similar to our approach for somatic and germline cancer models, we quantified the relative contribution of various features to the predictability of our rare and common disease cohorts. In contrast to our cancer models, we observed that the overlap of SVs with TAD boundary annotations had the most significant contribution in determining pathogenicity scores for SVs belonging to the CVD cohort (Additional file 2: SI Fig. S13). This observation is consistent with previous studies highlighting the role of germline SVs in various diseases through disruption of the three-dimensional genome structure. We note that TADs are highly conserved across multiple tissues and cell types [30]. Thus, upon replacement of the TAD definition (based on a different cell type) in our original CVD model, we observed a similar contribution of TAD annotation to the predictability of the modified model (Additional file 2: SI Fig. S14). Similarly, we observed higher contributions from the overlap of CTCF boundaries, sensitive regions, and SV length to our IBD germline model's predictive performance (Additional file 2: SI Fig. S15).

### Model evaluation: somatic models and gene enrichment analyses

**Fig. 3:**
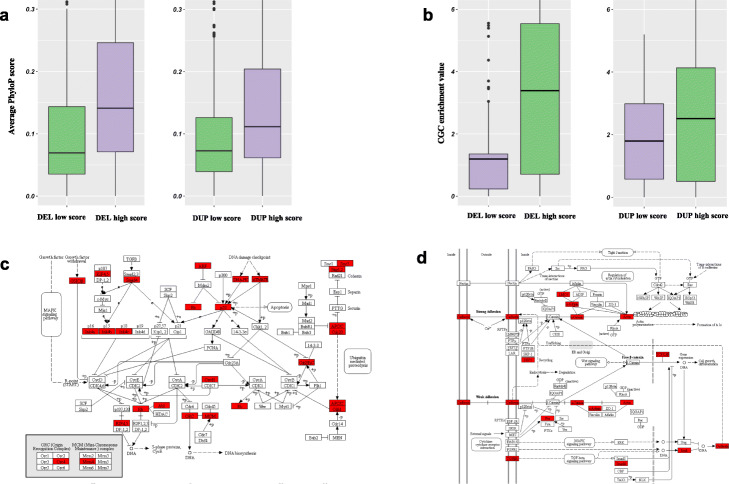
Orthogonal biological validations of somatic models in cancer. **a** This plot presents a mean conservation score comparison for genomic regions that overlap with predicted highly pathogenic deletions against benign deletions (left panel) and duplications (right panel) for a model where conservation was excluded from the original model. **b** This plot presents cancer gene enrichment values for coding regions that overlap with predicted highly pathogenic deletions against benign deletions (left panel) and duplications (right panel) for a model where overlap fraction with cancer genes was excluded from the original model. **c** Example showing the ubiquitin-mediated proteolysis pathway that is enriched among genes affected by highly pathogenic deletions on the pan-cancer level. Genes that are influenced by highly pathogenic deletions in this pathway are highlighted in red. **d** Example showing the adherens junction pathway, which is enriched among genes affected by highly pathogenic duplications on the pan-cancer level. Genes that are influenced by highly pathogenic duplications in this pathway are highlighted in red

In addition to accuracy assessment, we performed various analyses to investigate the biological validity and robustness of our approach for quantifying the pathogenicity of cancer SVs. For instance, we carried out a separate round of investigations in which we excluded cross-species conservation scores and overlap fractions with ultra-conserved and sensitive regions from our models. We computed the pathogenic score of each SV using these modified models and correlated them with the average PhyloP score for genomic regions overlapping with the SV. Relative to low pathogenic score SVs, somatic SVs with higher scores should intuitively overlap with more conserved regions of the genome. Indeed, we observed that highly pathogenic (SV pathogenic score ≥ 0.9) deletions and duplications exhibited higher conservation scores compared to benign deletions and duplications (SV pathogenic score ≤ 0.2). This observation was highly significant for both deletions and duplications (Fig. 3a).

Next, we assessed whether our machine learning approach assigns a high pathogenic score to SVs that are enriched among known cancer genes. As with the analysis detailed above, we excluded the known cancer gene overlap as a feature to generate modified random forest models for this analysis. We recomputed the pathogenic score for each SV using these modified models, and then classified SVs as high- and low-pathogenic SVs based on the thresholds detailed above. Subsequently, we quantified the enrichment of known cancer genes in the high- and low-pathogenic SV groups. As expected, we observed more substantial enrichment of known cancer genes among highly pathogenic deletions and duplications compared to those with lower pathogenic scores (Fig. 3b). As with our conservation analysis, the differences in enrichment between these SV groups were highly significant.

Finally, we used our original model to identify highly pathogenic deletions and duplications (SV pathogenic score ≥ 0.9) on the pan-cancer level. We then identified all coding genes that entirely or partially overlapped with these pathogenic deletions and duplications. Using this overlapping gene list, we performed ontology and pathway enrichment analysis. We found that pathogenic deletions influence genes that are enriched for vital biological processes, including signal transduction, cell cycle progression, post-translational modification, and DNA repair. Pathway-level analyses indicated that these pathogenic deletions affect critical pathways that involve Wnt and Ras signaling, cellular senescence, transcriptional regulation, and ubiquitin-mediated proteolysis (Additional file 2: SI Fig. S16, Additional file 1: Table S3-4). As an example, we highlight genes that are deleted by highly pathogenic SVs and are involved in the ubiquitin-mediated proteolysis pathway (Fig. 3c). Our results are consistent with prior studies that have shown that disruption of these pathways can drive tumor progression [31].

Similarly, highly pathogenic duplication influenced genes that are enriched for cell differentiation, development, signal transduction, and various metabolic processes. Pathway-level enrichment analysis suggested that such duplicated genes play a pivotal role in tyrosine receptor kinase signaling, post-translational protein modifications, membrane trafficking, and the Wnt signaling pathway (Additional file 2: SI Fig. S17, Additional file 1: Table S5-6). We highlight a set of genes—including those encoding cadherin, actin, SMAD4, DEP, and TGF beta receptor—that are affected by highly pathogenic duplication and play a vital role in the adherens junction pathway (Fig. 3d). The adherens junction pathway maintains homeostatic cell signaling, and its disruption is known to drive breast cancer progression [32].

### Model evaluation: comparison with a previous method and long-read analyses

We also compared our data-driven approach with the prior SVScore method [14] that assigns deleteriousness scores to SVs by applying a pre-computed base-pair score (measured for assessing the impact of single-nucleotide changes). We found that our SVFX method performed significantly better than SVScore in identifying pathogenic SVs in both somatic and germline contexts. For instance, in our independent testing dataset for various cancer cohorts, our somatic deletion model had better precision and recall (average auROC value of 0.84) compared to SVScore (an average auROC of 0.73) across multiple cancer cohorts (Additional file 2: SI Fig. S18a). Similarly, for the germline ClinVar [16] SV model, our approach showed significantly better performance (auROC of 0.99) compared to the SVScore method (auROC value of 0.9) in the independent testing dataset (Additional file 2: SI Fig. S18b). These observations further highlight the efficacy of our approach for detecting pathogenic SVs.

In recent years, there has been increasing interest in characterizing accurate SV maps for human genomes using long-read sequencing platforms. Our method is highly flexible and can be easily applied to SVs identified using long-read sequencing data. Despite the relative superior performance of long-read sequencing platforms in identifying SVs, we currently lack a large-scale SV dataset based on these approaches. Thus, as a proof of principle, we applied our method on deletions identified using long-read sequencing data for a breast cancer cell line [33] and “benign deletions” identified in healthy individuals [34]. We note that the number of samples in these datasets is relatively limited. However, we observed similar performance for our long-read germline deletion model compared to those using deletions based on short-read sequencing data (Additional file 2: SI Fig. S19). Overall, for the tenfold cross-validation (auROC value of 0.74) and the holdout testing datasets (auROC value of 0.7), our model showed good performance.

### Case studies highlighting high-impact deletions and duplications

Our machine learning framework can clearly distinguish between pathogenic cancer SVs and benign SVs. Based on the pathogenicity score, we highlight examples of somatic deletions and duplications that are predicted to be highly pathogenic in different cancer cohorts. Overall, we found that many deletions and amplifications with high pathogenic scores overlapped with regulatory regions in the genome. To visually inspect the effect of these variants, we used the H3K27ac histone modification from multiple tissues generated by the Roadmap Epigenome Mapping Consortium [19]. This particular histone modification marks the presence of cis-regulatory elements such as promoters and enhancers in the genome. We found that these example SVs influence regulatory elements that are active in multiple tissues, as reflected in the conserved H3K27ac signal profiles. Presumably, these conserved regulatory elements play an essential role in gene regulation, and thus their disruption through deletion or duplication is likely to be highly pathogenic. Moreover, we used the Hi-C contact matrix to inspect the chromatin structure around these deletions and amplifications [35].

**Fig. 4:**
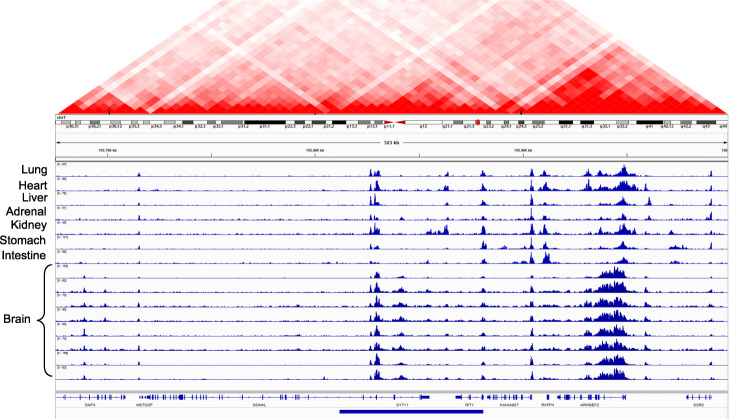
Example of a highly pathogenic cancer deletion that influences coding and regulatory elements in the genome. This figure presents a highly pathogenic deletion that disrupts entirely or partially the coding and regulatory regions of three distinct genes: *RIT1*, *SYT11*, and *GON4L*. The regulatory elements are marked by peaks observed in the histone mark (H3K27ac) signals across multiple tissues. The Hi-C matrix plot shows the TAD boundaries disrupted by this deletion

As an example, Fig. 4 shows annotation of a high-impact deletion that is also recurrent across multiple cancer types. As expected, this particular deletion overlapped with several noncoding elements, completely engulfing two genes (SYT11 and RIT1) and partially overlapping with the first exon of another gene (GON4L). The RIT1 gene encodes for a protein that plays a crucial role in the RAS/MAPK pathway and regulates the cellular signals required for cell proliferation and differentiation. The RIT1 gene belongs to the RAS family of oncogenes. A previous study [36] reported an association between RIT1 gene inactivation and lymphoma progression. Similarly, the GON4L gene is a transcription regulator that plays vital roles in cell division and differentiation. In particular, GON4L gene-based transcription regulation is essential for B cell development and differentiation [37]. Finally, the SYT11 gene encodes a protein that facilitates calcium signal-dependent membrane trafficking. In addition to affecting the coding regions of these genes, this particular deletion engulfs many cis-regulatory elements and thus influences their long-range interactions. In particular, we found that a deleted enhancer disrupts numerous three-dimensional interactions (shown by the Hi-C contact matrix above) within the vicinity of the locus.

**Fig. 5:**
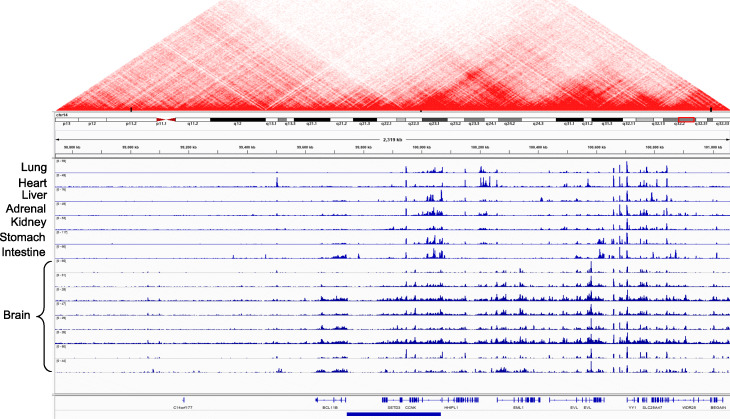
Example of a highly pathogenic cancer duplication that influences coding and regulatory elements in the genome. This panel presents a highly pathogenic duplication that influences coding and regulatory elements for multiple genes, including *BCL11B*, *SETD3*, *CCNK*, and *HHIPL1*. Similar to pathogenic deletion Fig. 4, this panel also displays the Hi-C profile to highlight TAD boundaries that are disrupted by this highly pathogenic duplication

We also describe an example of a highly pathogenic somatic duplication (Fig. 5) that directly overlapped with the SETD3 gene, a histone methyltransferase that is implicated in many diseases, including cancers [38]. The SETD3 gene also plays roles in cell cycle regulation, cell death, and chromosomal translocation [39, 40]. Furthermore, the overexpression of SETD3 leads to cell proliferation and tumor growth in liver cancer cells [41]. In addition to SETD3, this particular amplification affects the CCNK gene, which plays a vital role in transcriptional regulation. The chromatin structure from Hi-C data showed interactions of the regulatory element affected by this amplification with nearby genes that include YY1. The YY1 gene plays a dual role in activating and repressing of a large number of gene promoters. Overall, both of these examples highlight the efficacy of our approach in identifying highly pathogenic SVs. Furthermore, they provide essential insights into higher-order regulatory interactions that are affected by some of these variants.

Finally, we present a few examples of highly pathogenic germline deletions in the CVD and IBD cohorts. For instance, one of the predicted highly pathogenic deletions in our CVD cohort overlaps with the coding region of the *Clcn3* gene, which encodes the voltage-gated chloride channel protein CLC-3 (Additional file 2: SI Fig. S20). A previous study showed that the deletion of Clcn3 potentially affects the ion channel involved in cell volume homeostasis, which likely drives the development of myocardial hypertrophy and heart failure [42]. Similarly, we identified a putative pathogenic deletion in the IBD cohort that is proximal to the promoters of the *SLC23A2* gene (Additional file 2: SI Fig. S21), which has essential roles in vitamin C metabolism that are directly associated with the manifestation of IBD [43]. This deletion overlaps with weak enhancer sites and boundaries of two large TADs, which can be potentially deleterious to the proper regulation of the surrounding genes. These examples further highlight the ability of our method to detect biologically relevant pathogenic SVs in common disease cohorts.

## Discussion

One of the fundamental goals of population-level [17] and disease-specific [15, 29, 44] sequencing studies has been to identify causal SNPs and indels in a large pool of candidate variants. Such efforts have resulted in multiple tools and metrics to prioritize SNPs and indels. However, large SVs represent an essential set of variations that influence the linear as well as the three-dimensional genome structure. These alterations can perturb protein-coding regions and cis-regulatory elements alike, and they are often involved in various diseases, including cancer [7]. Despite altering a considerable fraction of the genome, there have been relatively few systematic studies to prioritize and identify pathogenic SVs. A lack of such efforts can be partially attributed to challenges associated with the accurate identification of SVs and their precise breakpoints using short-read sequencing technologies [8]. However, with the development of better tools and methods for SV discovery using short- and long-read techniques [8], we anticipate that generating a high-resolution map of genomic rearrangements will soon become routine. Thus, new methodologies for evaluating the pathogenicity of SVs are needed.

In this work, we present a new machine learning-based framework to assess the pathogenicity of SVs in disease cohorts. We note that our SV pathogenicity score quantifies the probability of given SVs belonging to a particular disease cohort compared to being present in a healthy population. Thus, one could interpret our models as disease classifiers rather than frameworks that explicitly quantify the endophenotypic effect of a given SV. Although we applied our method to a handful of cancer, common (CVD and IBD), and rare disease contexts, it could be easily extended to other disease studies. Overall, our method accurately identifies highly deleterious SVs and distinguishes them from low-scoring benign SVs. For somatic deletions and duplications in cancer, our models performed very well with mean auROC values of 0.865 and 0.835, respectively. We observed small differences in the predictive performances among different cancer cohort models, which can be related to the underlying differences in sample size (influencing the number of SVs). Likewise, our cancer germline deletion model was highly accurate (with a mean auROC of 0.8) across six cancer cohorts. Additionally, the auROC values of the germline models for different cancer cohorts were remarkably similar. We expect that including additional high-quality common SVs in our training dataset will further improve the discriminative performance of the germline model. Finally, we applied our framework to assign a pathogenic score to germline SVs in common and rare disease contexts. For instance, our germline models for CVD (mean auROC of 0.76) and IBD (mean auROC of 0.73) cohorts achieved high predictive accuracy, comparable to that of germline cancer models. Notably, our model trained on the ClinVar SV database achieved near-perfect accuracy (auROC of 0.99) compared to the previous SVScore method (auROC of 0.9). These results further highlight the applicability of our approach in different disease contexts.

We also built multiple versions of our original somatic models to evaluate the biological validity and robustness of our approach. For instance, we applied a truncated somatic model (by excluding conservation score and related annotations) to show that pathogenic SVs overlap with highly conserved regions in the genome. Similarly, we built another version of our original model in which we removed annotations for known cancer genes. We found that high-scoring SVs identified from these models were significantly enriched among known cancer genes compared to low-scoring SVs. These observations further suggest that our machine learning framework is robust and assigns biologically intuitive pathogenic scores. Finally, we performed pathway and ontology enrichment analyses of genes that overlapped with high-scoring SVs, as identified by our original model. We observed an enrichment of genes in many cancer-related pathways, including Wnt signaling, Ras signaling, DNA repair, cell differentiation, and ubiquitin-mediated proteolysis. These results further support the biological validity of our approach for assigning pathogenic scores to cancer-associated SVs.

As noted above, our machine learning framework is flexible and can be easily extended to assign pathogenic scores for SVs in whole-genome studies on other diseases, including autism and neuro-developmental diseases. Additionally, our current framework primarily focuses on identifying pathogenic deletions and duplications. However, it can be readily extended to detect pathogenic inversions and translocations in these diseases. We note that, currently, there is a lack of high-quality inversions and translocations in public databases, including 1KG [45], gnomAD [46], and ClinVar SV databases [47], limiting the applicability of our approach toward distinguishing all disease-associated SVs from benign ones. However, the rapid rise in long-read base sequencing platforms and their inherent ability to resolve inversions, translocations, and complex SVs will facilitate the generation of comprehensive SV resources. In the future, we can employ such extensive resources to train somatic and germline models to detect highly pathogenic inversions and translocations from benign ones.

In summary, despite the crucial role of SVs in various diseases, few approaches are currently available for interpreting and prioritizing these variants. At a per-nucleotide level, SVs contribute far more substantial variation in an individual genome than other mutations. However, SVs are often neglected as a consequence of the technical challenges associated with their identification and interpretation. We addressed this challenge by building a new framework that utilizes tissue-specific genomic and epigenomic features to quantify the pathogenicity of SVs. Identifying such pathogenic SVs along with deleterious point mutations and indels will facilitate a more complete understanding of the biology of various diseases.

## Methods

### Model construction and validation

SV coordinates were first gathered from the PCAWG project [15, 28] and 1KG SV datasets. 1KG SVs were assumed to be benign, and the variants from the cancer source, while not all deleterious, were expected to contain some set of harmful variants, which we aimed to identify through our method. We followed a similar approach for germline SVs. However, for cancer germline SVs, we utilized common 1KG SVs (AF > 0.5%) as benign variants. In contrast, for the CVD and IBD cohorts, we leveraged SVs present in the control group and gnomAD as the benign variant set, respectively. Moreover, we also utilized pathogenic and common benign SVs present in the ClinVar database to train and assess the performance of the germline models. Furthermore, to assemble these disease and benign SV datasets, we also considered the underlying SV types. For instance, our somatic deletion models include only deletions (with length > 50 bp) that are present in the cancer cohort and 1KG SV datasets. Similarly, our duplication models are comprised of duplications that are present in the disease and corresponding control/benign cohorts. Overall, the number of deletions was significantly higher than duplications in our disease and corresponding benign datasets (Additional file 1: Table S7). For instance, our somatic dataset included 7295 deletions on average (maximum and minimum frequency of 11,917and 4514 in BRCA and STAD cohorts, respectively) across all six cancer cohorts. In contrast, there were 5204 duplications on average (with maximum and minimum of 6023 and 3675 for BRCA and SKCM cohorts, respectively). Similarly, our germline dataset included, on average, 9094 deletions across multiple cancer types and 9306 deletions in the CVD cohort [48].

As expected, we observed significant disparity in the length distribution for SVs in our disease cohort and control datasets, especially in the somatic context (Additional file 2: SI Fig. S1). These length differences are likely to influence implicitly various features necessary to train our machine learning model. Thus, to avoid the effect of such length bias on our models, we generated random SV coordinates for both disease and benign datasets. For each original SV (deletion or duplication) in the disease and benign set, we uniformly sampled one thousand randomized SV instances. Each of the randomized SVs had the same length and occupied the same chromosome as the original SV. Along with length distribution disparity, differences in the number of SVs between the disease and benign cohorts could bias our models. To address this challenge of data imbalance, we randomly subselected SVs from the 1KG SV and other control datasets such that the number of SVs belonging to the benign/control dataset equaled the corresponding number of disease-associated SVs. We applied this data balancing approach to both our somatic and germline models.

For each SV (identified solely by coordinates), a variety of features were calculated and compiled into a feature matrix. Three categories of features were selected: tissue-specific functional genomics data, various annotation metrics (Additional file 1: Table S1), and conservation scores. For example, we obtained different tissue-specific histone mark (H3K27ac, H3K4me1, H3K4me3, H3K36Me3) signals from the ENCODE [49], IHEC [50], and Epigenome Roadmap project [19]. Similarly, we downloaded genome-wide methylation, GC content, CTCF, and replication timing data from the ENCODE project. For genomic annotations, we utilized Gencode v.19-based definitions of coding regions, splice sites, promoters, and UTRs. Furthermore, we used the “collapsed version” of the original genomic annotations to address complexities due to the presence of multiple transcripts for a gene. These collapsed annotations were defined by taking the union of a given genomic element for all individual transcripts. Moreover, we employed additional annotations, including fragile site regions, SINE elements, TADs, heterochromatin regions, and known cancer genes. Finally, we used multiple conservation-related features to train our models. These features included 100-ways cross-species PhyloP scores, annotations for ultra-conserved and sensitive regions across the genome.

Annotation overlaps were calculated as the percentage of the variant that overlapped with any region in a given annotation dataset. For example, given a 10,000-nucleotide variant and a set of coordinates corresponding to TADs, if 5000 of the nucleotides in the variant were within one of the TADs, then the overlap metric would be 0.5. For tissue-specific epigenomic and functional genomics data-based features, we divided SVs into windows of 10 bp length and computed the features over these windows. For instance, given an SV [*a*, *b*] that starts at genomic position *a* and ends at position *b*, we divided the interval into 10-bp bins, i.e., $n=\frac{b-a}{10}$ bins. For the *i*th bin (*n* ≥ *i* ≥ 1), we computed the total signal values for each functional genomics and epigenomic dataset within the bin. Subsequently, we calculated the average of these values for each dataset over all 10-bp bins that overlap with a given SV. We applied this 10-bp bin approach to increase the efficiency of our computation. Furthermore, this method provides us flexibility such that one could utilize the maximum or minimum of these bins as a feature instead of using the mean value applied in the current study. Combined, the total set of features can be summarized as:

$$ SV\left[a,b\right]\to \Big({\overline{F}}_{H3K27 ac},{\overline{F}}_{H3K4 me3},{\overline{F}}_{H3K36 me3},{\overline{F}}_{H3K27 me3},{\overline{F}}_{H3K4 me1},{\overline{F}}_{gcContent}, $$

$$ {\overline{F}}_{repTiming},{\overline{F}}_{CTCF},{\overline{F}}_{WGBS},{\overline{F}}_{PhyloP},{F}_{Annotation},{F}_{Cosmic}\Big) $$

where $\overline{s}$ denotes the average signal over all the 10-bp bins within [*a*, *b*]. Overall, we computed 22 features and used them to build the model. As discussed earlier, we used these features because prior studies have shown strong correlation with a subset of these features and distribution of SVs in the genome [23–27]. Additionally, our annotation-based features are likely to capture properties of the coding and noncoding functional elements in the genome.

After extracting the relevant features for both disease-associated and benign SVs, we normalized the original feature matrix. The feature normalization was essential to avoid any implicit feature bias due to length distribution differences observed between disease and benign SV datasets. For a given feature, we computed the feature value of each original SV and the corresponding values for the thousand randomly shuffled SV instances of that particular SV. Subsequently, we applied a *Z*-score transformation to perform the normalization for the disease-associated and benign SV dataset. Moreover, we assigned class labels (1 for all variants from the disease dataset, and 0 for 1KG/control SVs). While all disease SVs are not deleterious (in fact, only a minority were expected to be), our rationale behind the labeling method was that benign variants would mostly share characteristics with 1KG SVs. Thus, our model would only predict a variant’s class to be 1 with high confidence if the variant was very different from a benign variant. Finally, we appended the length of SVs in *Z*-score-transformed feature matrices for training and testing the machine learning models. The explicit inclusion of length feature is needed to clearly distinguish large-size SVs in the somatic and germline context. We note that a small set of features for a subset of SVs in our training and testing dataset for various disease cohorts consist of missing value. For such features, we adopted a simplistic approach by assuming that these features for a given SV manifest no difference between disease and the corresponding healthy cohorts. Thus, for a given SV, we assigned a value of zero for such features in the *Z*-score-normalized feature matrix.

Once the feature matrix was compiled and normalized, the data was used to train ten random forest models. Each model was trained on a disjoint 10% of the data. Then, each model predicted a probability for the remaining 90% of the data for a class label of 1 (i.e., that the variant was from the disease-associated SV dataset). The nine probabilities for each variant were averaged to produce one final score, meant to reflect the probability that the variant was a member of the disease-associated dataset. Thus, by ordering variants by these scores, we could construct a ranking of variants. Variants with very high probabilities, near the top of the ranking, had characteristics that were very different from the set of “benign” 1KG/control dataset variants; by contrast, variants with low (around 0.5 and below) probabilities had features that were virtually indistinguishable from those of benign variants. We also performed hyper-parameter tuning to optimize different somatic and germline models using 70% of each disease and the corresponding healthy dataset. For hyper-parameter optimization, we systematically varied the maximum depth of each tree (value ranges between 2 and 10), the total number of trees in the forest (in the range of 10 to 5000), and the minimum amount of leaves required to split an internal node (in between 10 and 100). The primary considerations behind our choices of these hyper-parameters were interpretability, tractability, and model performance. Maximum depth and the number of trees in a forest are intuitive, necessary, and well-documented controls of model complexity in tree-based and forest-based models. In particular, tuning these two parameters helps navigate the bias-variance tradeoff for an ensemble-based tree model. Similarly, varying the minimum number of leaves required to split an internal node influences the model performance.

We applied training datasets for each disease/benign cohort to build a comprehensive set of models. Subsequently, we evaluated precision-recall values for each model to select the optimal one with the highest auROC and auPRC value. The source code for the SVFX workflow is available on the project’s Github page (https://github.com/gersteinlab/SVFX). The SVFX workflow is implemented in python3 and utilizes various python-based packages including pybigwig, scikit learn, and matplotlib.

### Downstream analyses

In order to perform orthogonal validation, we modified the original feature matrix to generate two modified models. In one such model, we removed the average cross-species conservation (PhyloP) score and the overlap fraction of SVs with ultra-conserved and sensitive regions in the human genome. Similarly, in a different model, we removed features capturing the overlap fraction of SVs with known cancer genes as defined in the cancer gene census database. For both of these modified models, we followed the same procedure of *Z*-score-based feature normalization, training, and testing. For the model without conservation score, we defined highly pathogenic SVs based on a pathogenicity score threshold above 0.9 and benign SVs with a pathogenicity score below 0.2. For pathogenic and benign classes of SVs, we then computed the average conservation score by taking the mean value of nucleotide-level PhyloP score for regions overlapping with a given SV.

Similarly, for the model without cancer gene annotation, we applied the same SV impact thresholds to classify SVs into benign and pathogenic groups. For each group of SVs, we computed the fraction of overlap between known cancer genes and member SVs for different cancer types. For enrichment calculation, members of the pathogenic and benign SV groups were permuted one thousand times across the genome. For each cancer gene, we computed the fraction of nucleotides overlapping with the original and permuted SVs to calculate a *Z*-score-based enrichment score. Subsequently, we compared these *Z*-score enrichment scores to measure differences between pathogenic and benign SVs. Finally, we calculated the gene ontologies and pathway enrichments of genes that partially or completely overlapped with highly pathogenic SVs. Pathway enrichment was done for KEGG as well as the reactome database.

Finally, we compared SVFX with the previously reported SVScore method that assigns functional impact score to SVs by leveraging genome-wide per-base score (developed initially for point mutations) [14]. We used the SVScore assigned based on the mean of per-base scores for nucleotides that overlap with a given SV region. SVFX assigns a normalized score to a given deletion or duplication. Thus, to compare SVFX and SVScore, we transformed the original SVScore value on a normalized scale. We note that, by default, SVScore assigns a score of 100 to SVs with a length higher than 1 mb. On this normalized scale, we assigned these SVs an SVScore value of one. We applied both SVFX and SVScore for independent testing of somatic deletion datasets for multiple cancer cohorts and 1KG SVs. Moreover, we compared the performance of the SVFX and SVScore methods on holdout testing datasets for germline ClinVar pathogenic and benign variants. This comparison evaluated the performance of both approaches for correctly identifying disease-associated deletions. Finally, we also report pathogenicity scores for deletions and duplications in the ClinVar database (Additional file 1: Table S8-9).

### Source code and data availability

All cancer-associated SV datasets analyzed in this manuscript are available to the community via International Cancer Genome Consortium- and The Cancer Genome Atlas-associated PCAWG data portals (https://dcc.icgc.org/releases/PCAWG/consensus_sv) using controlled data access [51]. The 1000 Genomes Phase 3 SV datasets [45] were downloaded from the 1000 Genomes Project data portal. Tissue-specific epigenomics and functional genomics data were downloaded from iHEC [52, 53], Epigenome Roadmap [54], and ENCODE project data portal [55]. SVs belonging to CVD [48] and IBD [56] cohorts were generated by the NHGRI centers for common disease genomics consortium. Finally, we also utilized SVs belonging to the ClinVar database [47]. The source code for SVFX workflow is available on the project’s Github page [57] (https://github.com/gersteinlab/SVFX) under MIT License. Finally, we also provide pre-trained somatic and germline models through the SVFX Github page. We note that these pre-trained models disallow the extraction of training SV coordinates to avoid any potential variant leakage.

## Supplementary information

**Additional file 1.** Supplementary datasets.

**Additional file 2.** Supplementary Figures.

**Additional file 3.** Review history.
