# Supplementary material for: SVFX: a machine learning framework to quantify the pathogenicity of structural variants
Source: Genome Biol. 2020 Nov 9;21:274. doi: 10.1186/s13059-020-02178-x (PMC7650198; doi:10.1186/s13059-020-02178-x)
Supplement: Supplementary file 2 — Additional file 2. Supplementary Figures. [file 13059_2020_2178_MOESM2_ESM.pdf]

Supplementary Figures

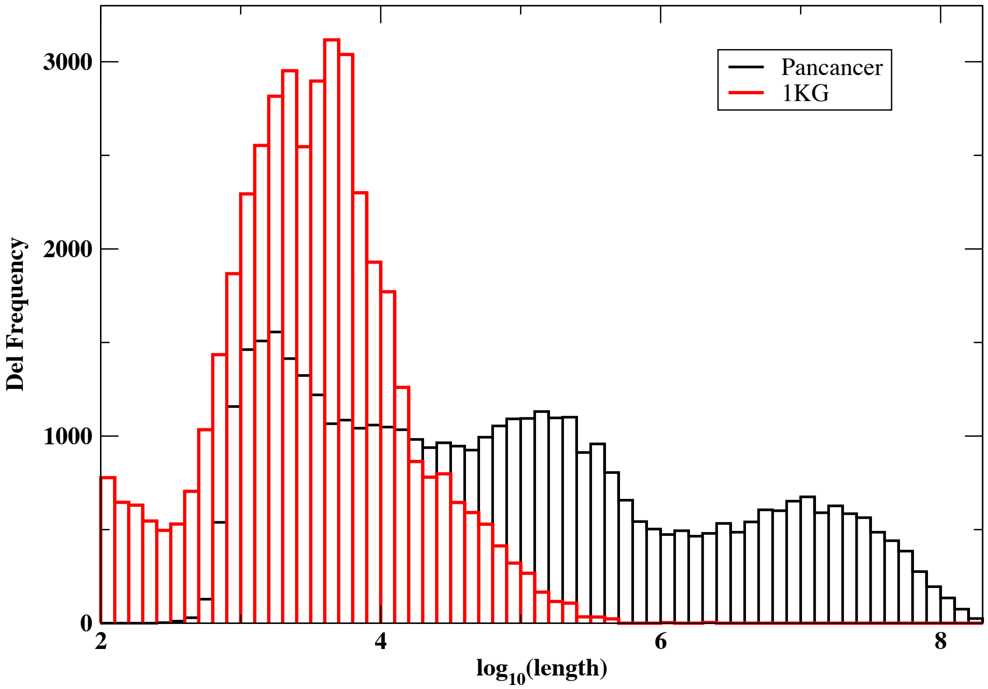

SI FIG S1: *length distribution for deletions in pan-cancer (six cancer) and 1KG cohorts*. Y-axis of histograms presents the number of SVs falling into a particular log-bin. Black and red histograms correspond to the pan-cancer and 1KG cohort, respectively.

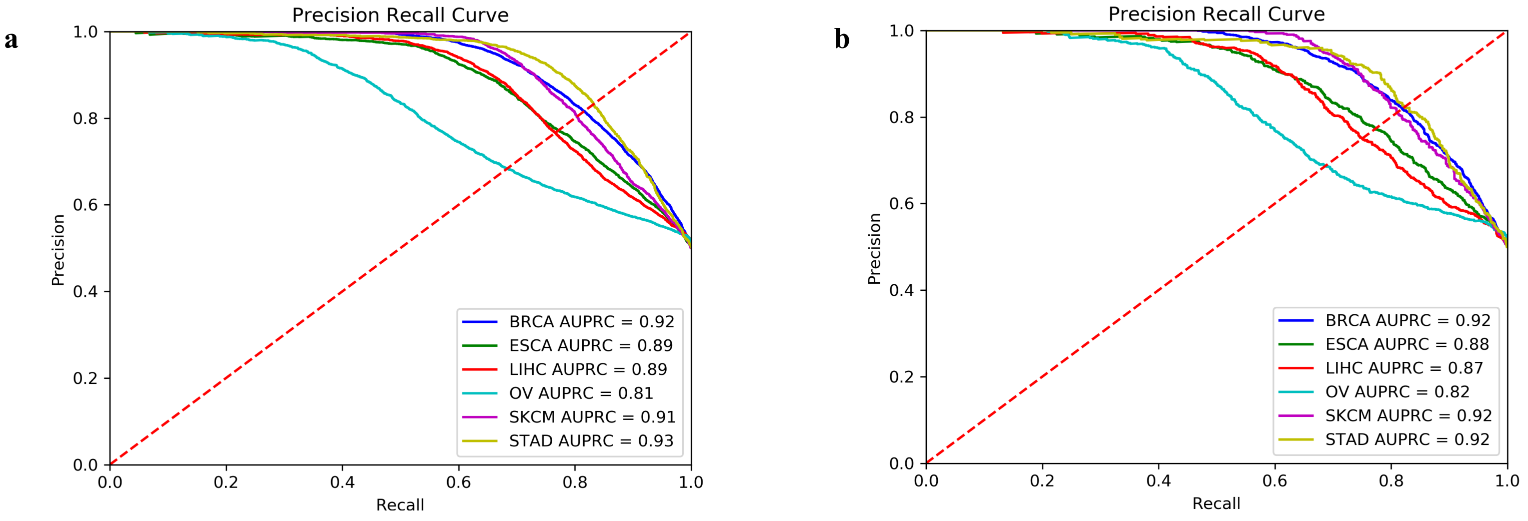

SI FIG S2: *Area under Precision Recall Curves(auPRC) for large somatic deletions belonging to six cancer cohorts using the validation and testing datasets*. a) auPRC plots for somatic DELs in six cancer cohorts using 10-fold cross validation approach, b) auPRC plot for somatic deletions in six distinct cohorts using independent testing datasets that were not used to train random forest models.

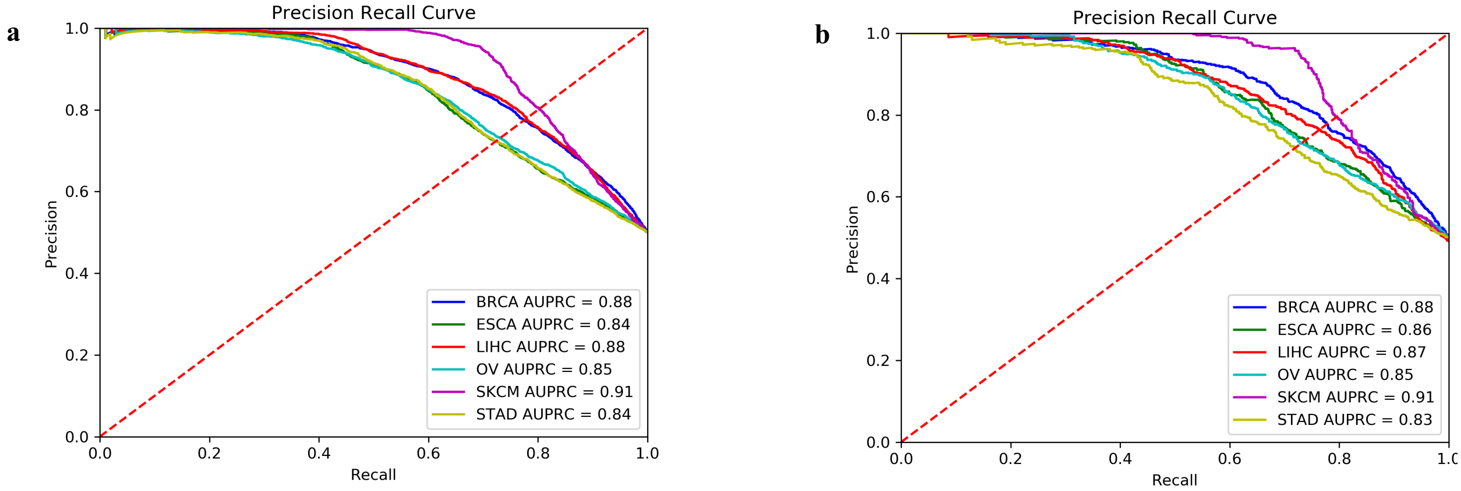

SI FIG S3: *Area under Precision Recall Curves(auPRC) for somatic duplications belonging to six cancer cohorts using the validation and testing datasets.* a) auPRC plots for somatic duplications in six cancer cohorts using 10-fold cross validation approach, b) auPRC plot for somatic duplications in six distinct cohorts using independent testing datasets.

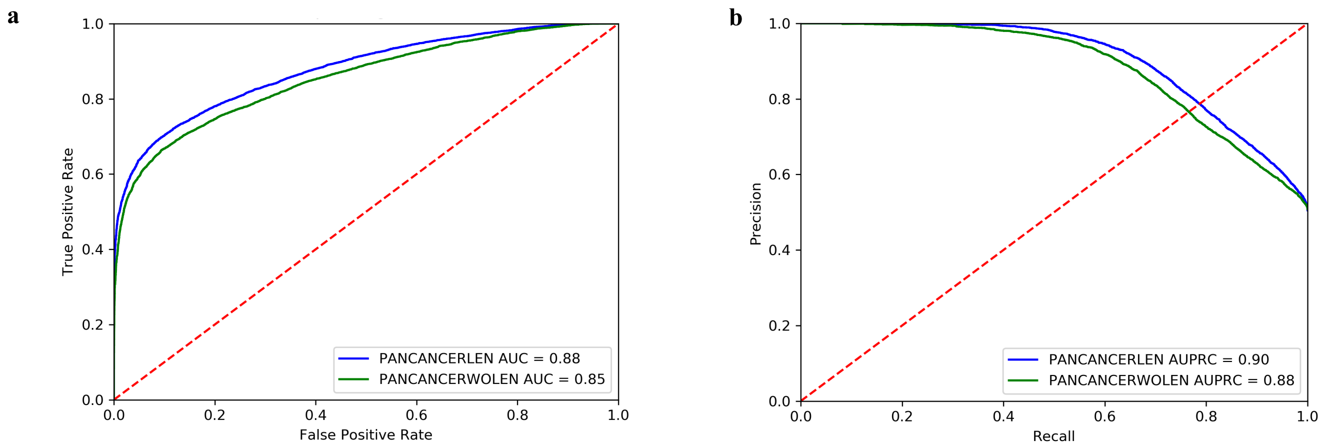

SI FIG S4: *Area under Receiver Operating Characteristics (auROC) and Precision Recall Curves(auPRC) for somatic deletions belonging to six cancer cohorts using model with and without length as feature.* a) auROC plots for somatic deletion on pan-cancer (aggregated over six cancer cohorts) level using testing dataset, b) auPRC plot for somatic deletion on pan-cancer (aggregated over six cancer cohorts) level using testing dataset. In both plot, blue and green curve correspond to model trained using feature matrices with and without length as explicit features, respectively.

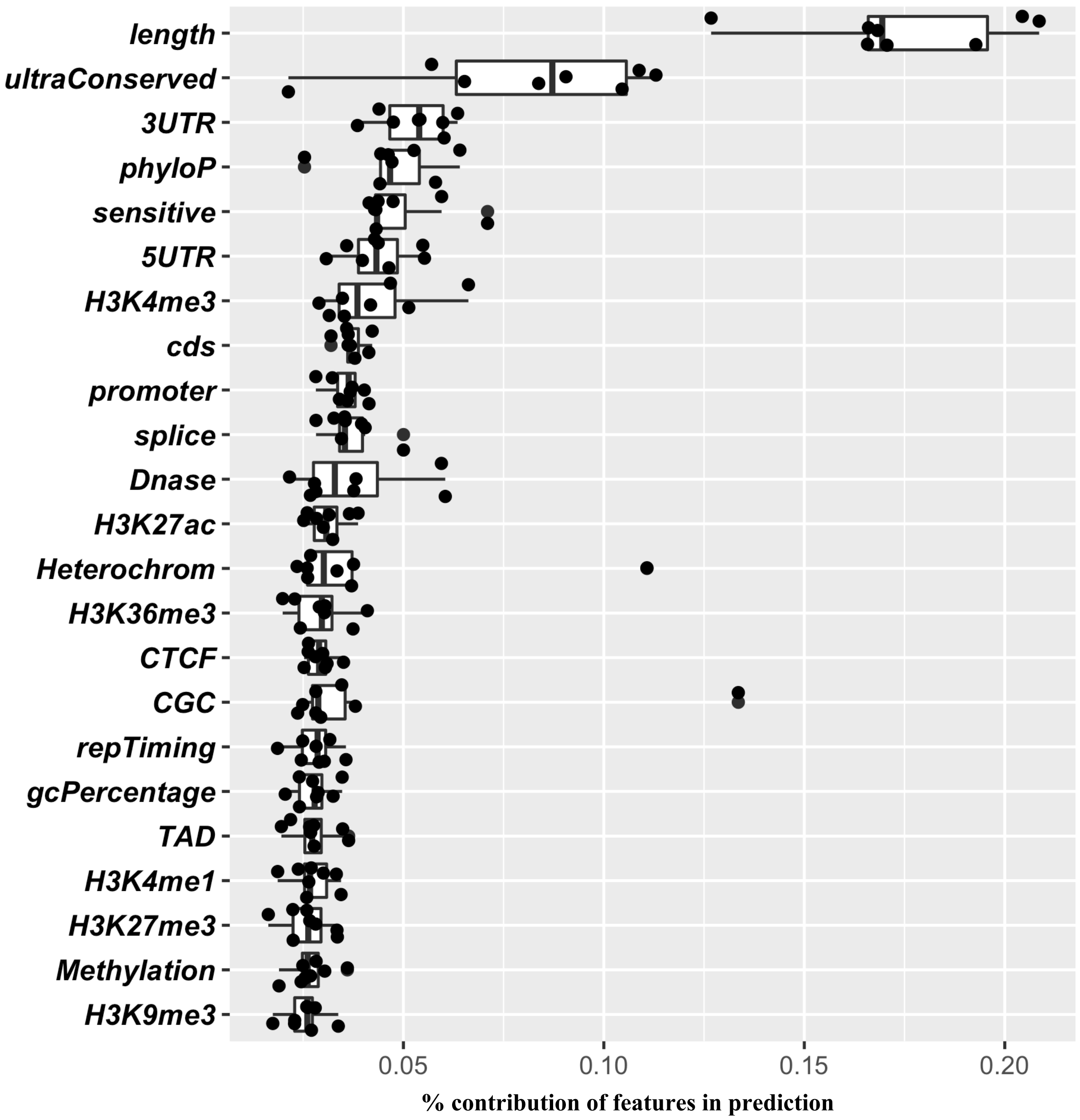

SI FIG S5: This Figure presents the percentage contribution for distinct features in the overall predictability of somatic deletion models for six cancer cohorts. Each data point in the boxplot corresponds percentage contribution of a feature in the overall performance in a cancer cohort.

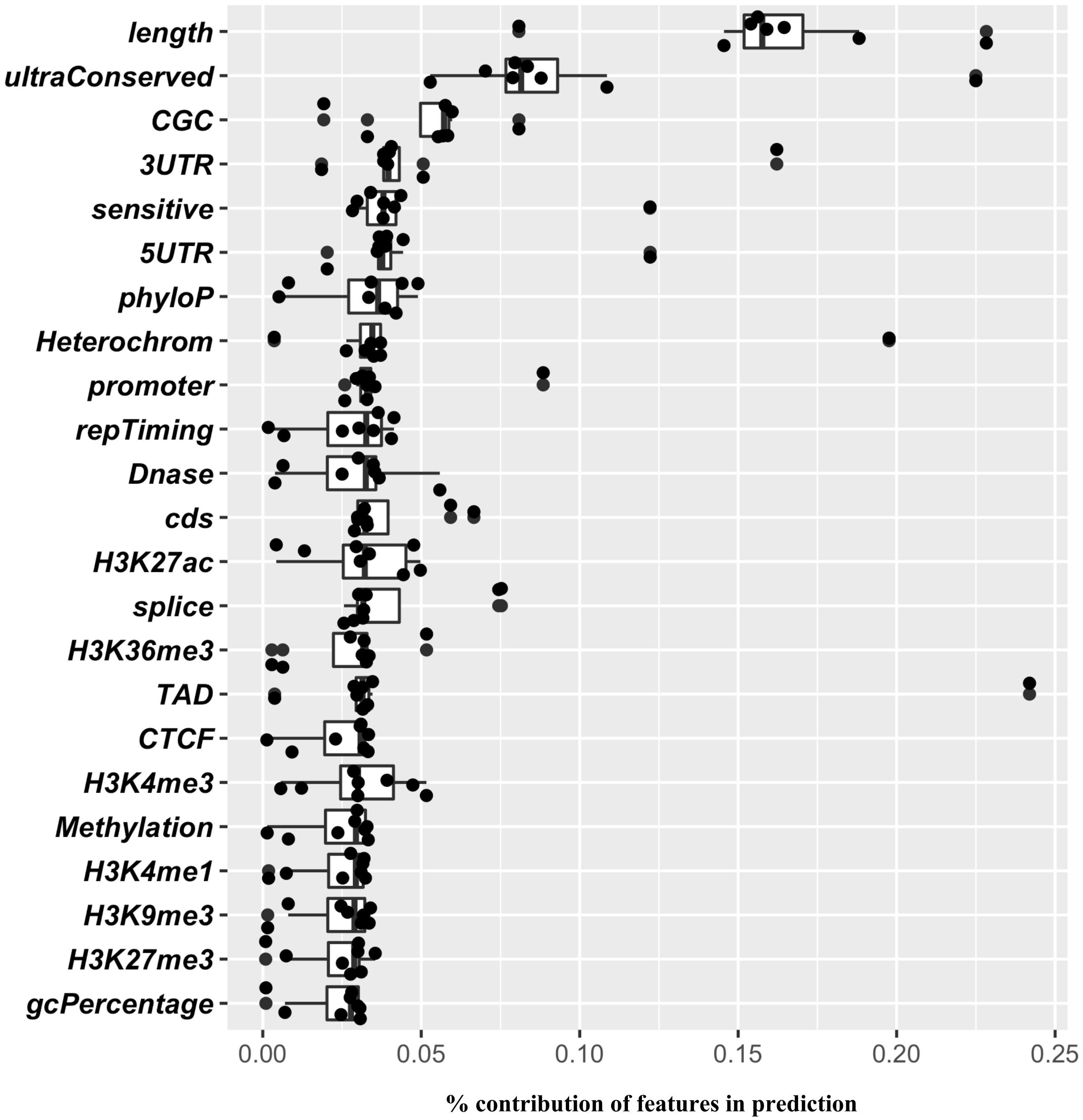

SI FIG S6: This Figure presents the percentage contribution for distinct features in the overall predictability of somatic duplication models for six cancer cohorts. Each data point in the boxplot corresponds percentage contribution of a feature in the overall performance in a cancer cohort.

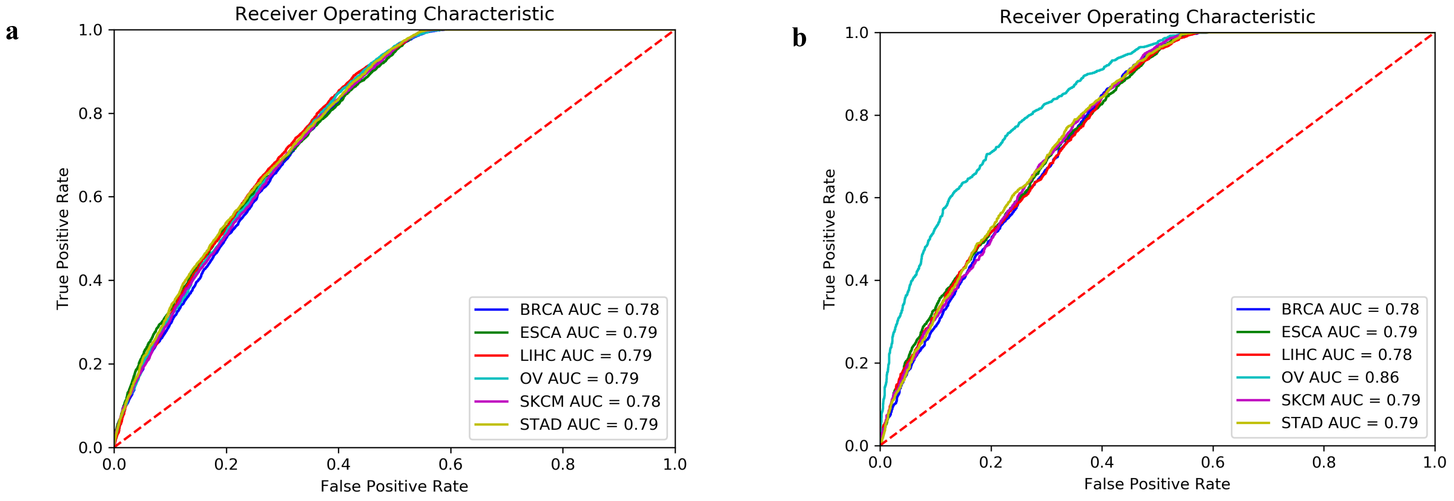

SI FIG S7: *Area under Receiver Operating Characteristics (auROC) for germline deletions belonging to six cancer cohorts using the validation and testing datasets.* a) auROC plots for germline deletions in six cancer cohorts using 10-fold cross validation approach, b) auROC plot for germline deletions in six distinct cohorts using independent testing datasets that were not used to train random forest models.

a

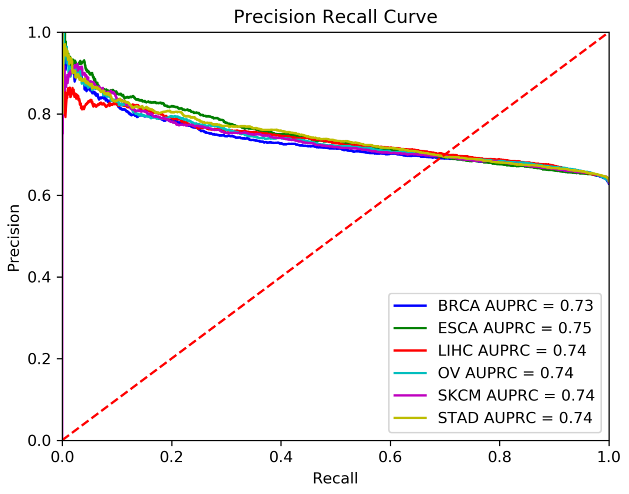

b

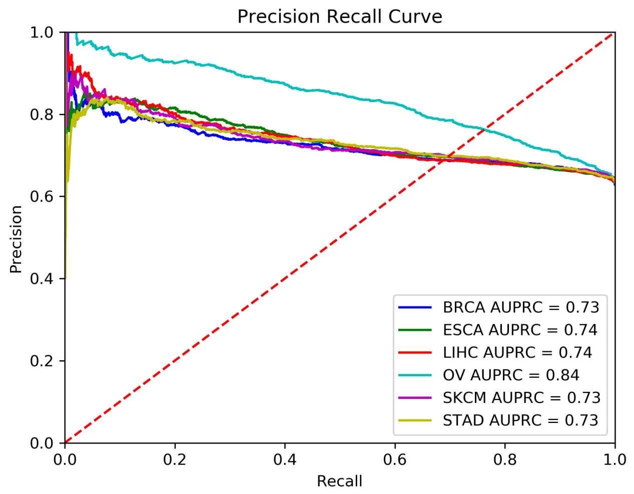

SI FIG S8: *Area under Precision Recall Curves(auPRC) for germline deletions belonging to six cancer cohorts using the validation and testing datasets.* a) auPRC plots for germline deletions in six cancer cohorts using 10-fold cross validation approach, b) auPRC plot for germline deletions in six distinct cohorts using independent testing datasets.

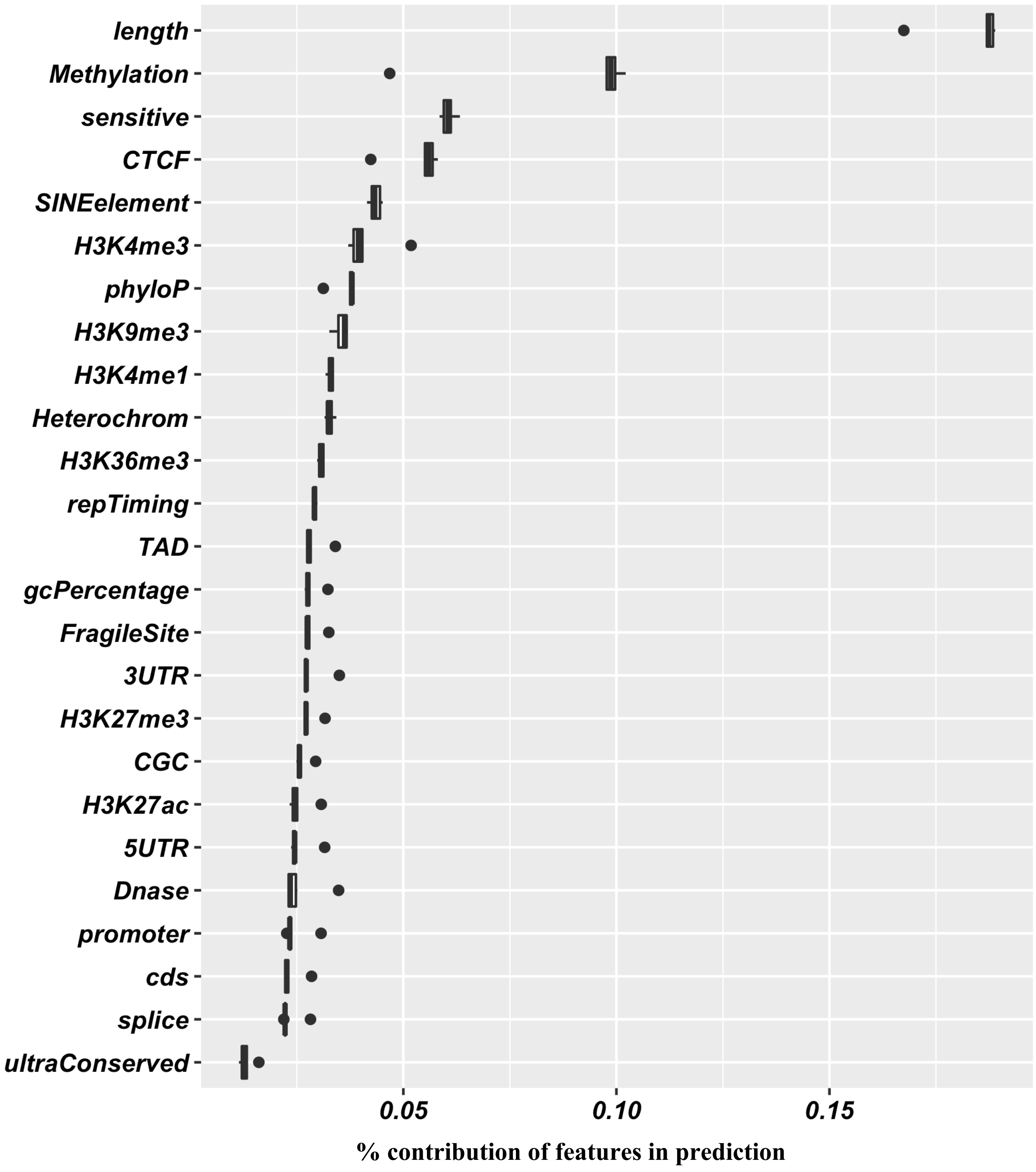

SI FIG S9: This Figure presents the percentage contribution for distinct features in the overall predictability of germline deletion models for six cancer cohorts. Each data point in the boxplot corresponds percentage contribution of a feature in the overall performance in a cancer cohort.

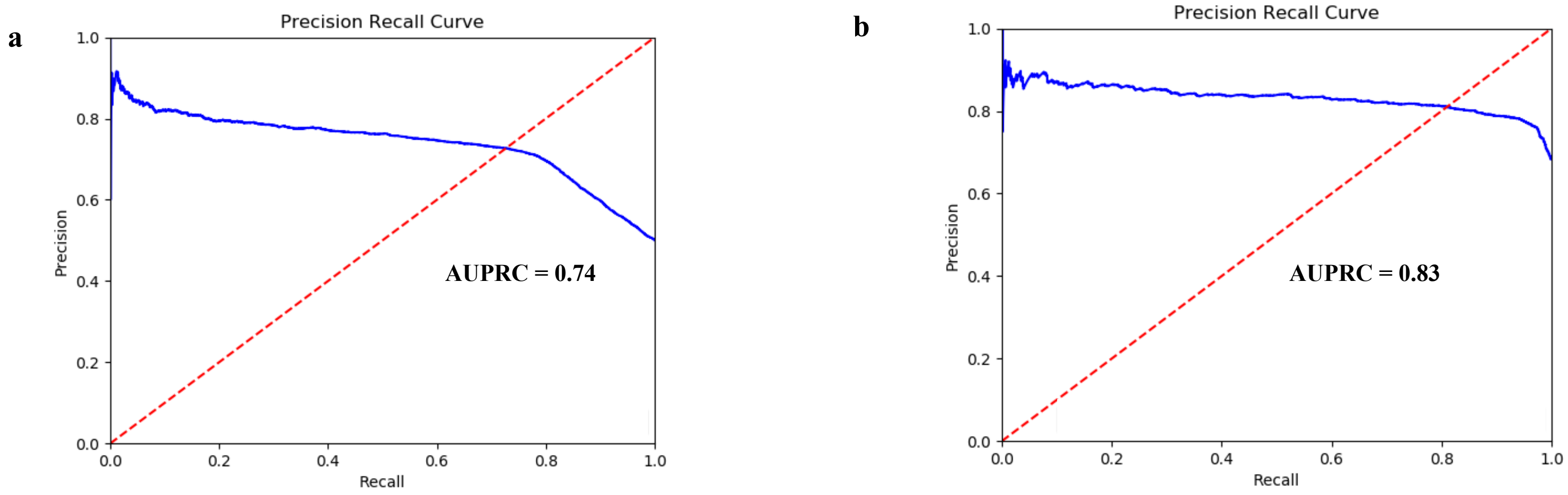

SI FIG S10: *Area under Precision Recall Curves(auPRC) for germline deletions in the cardiovascular disease cohort using the validation and testing datasets.* a) auPRC plots for germline deletions in the cardiovascular disease cohort using the 10-fold cross validation approach, b) auPRC plot for germline deletions in the cardiovascular disease cohort using independent testing datasets.

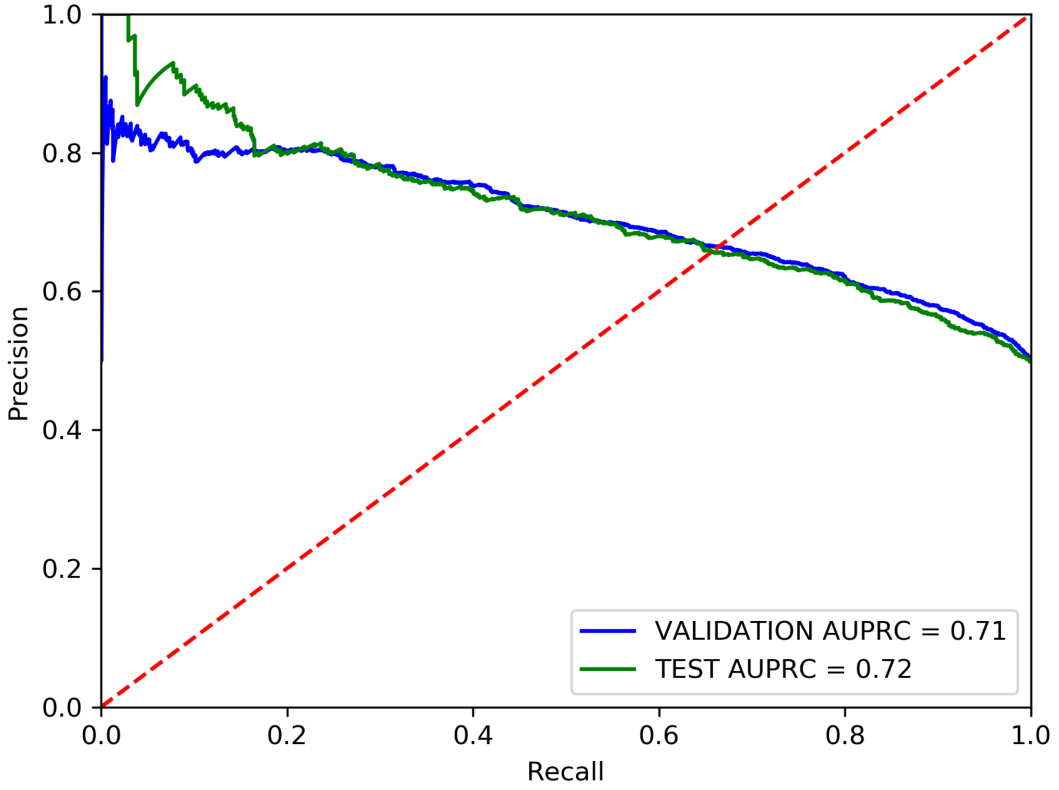

SI FIG S11: *Area under Precision Recall Curves(auPRC) for germline deletions in the inflammatory bowel disease cohort using the validation and testing datasets.* auPRC plots for germline deletions in the inflammatory bowel disease cohort using the 10-fold cross validation approach and independent testing datasets. Blue and green curve represent evaluation using 10-fold and independent testing dataset, respectively.

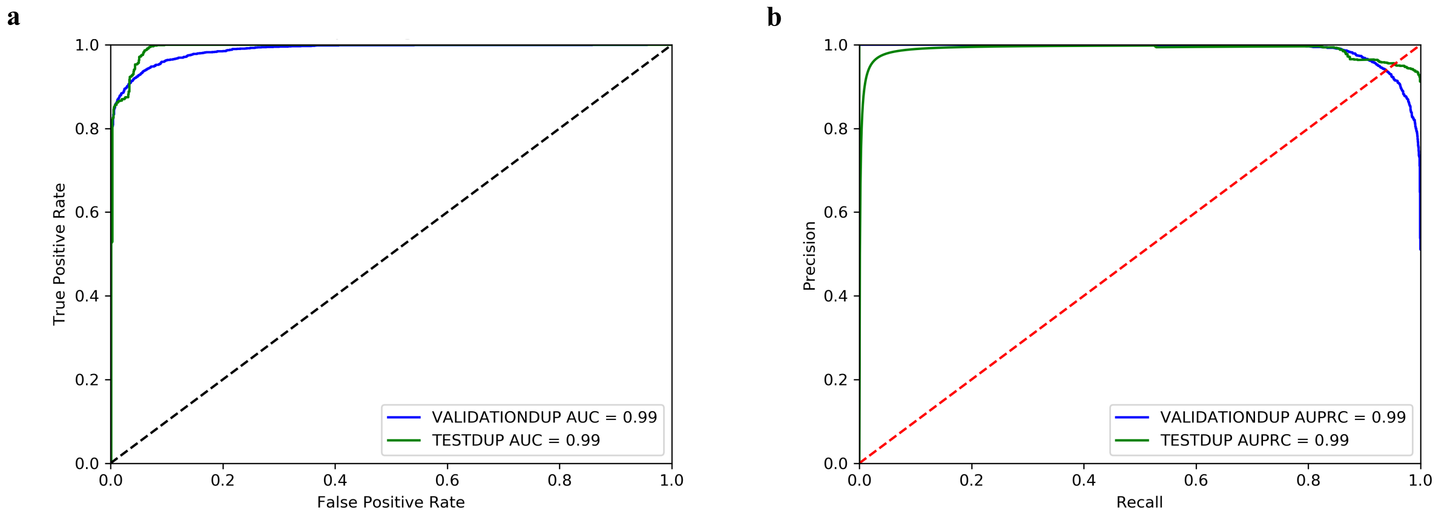

SI FIG S12: *Area under Receiver Operating Characteristics (auROC) and Precision Recall Curves(auPRC) for germline duplications in the ClinVar database.* a) auROC plots for germline duplications in the ClinVar database using the 10-fold cross-validation and independent testing dataset approach, b) auPRC plot for germline duplications in the ClinVar database using the 10-fold cross-validation and independent testing dataset.

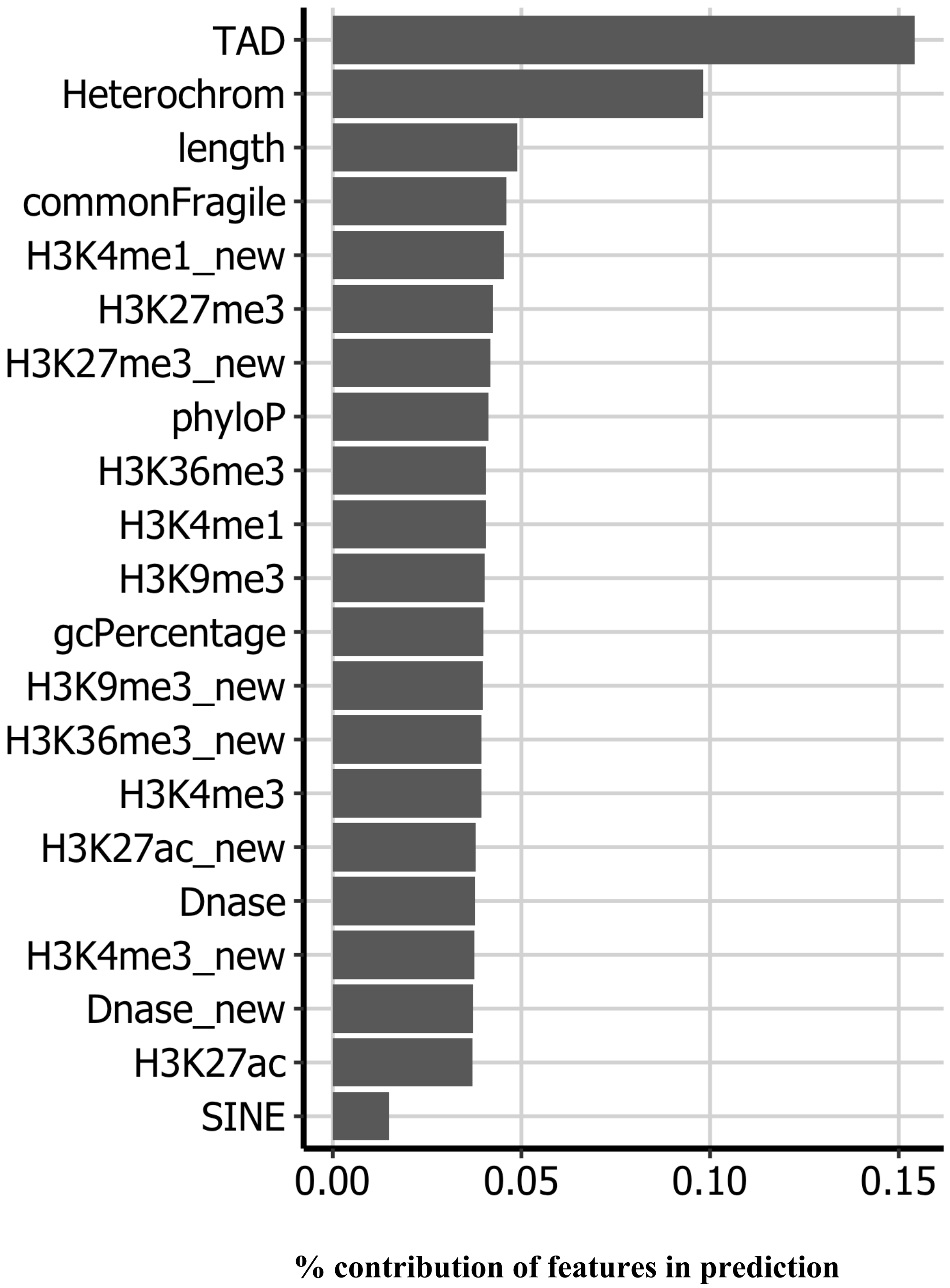

SI FIG S13: The above bar plot presents the percentage contribution for distinct features in the overall predictability of the germline deletion model for the cardiovascular disease cohort. We utilized IMR90 cell line-based TAD definition to build this particular model.

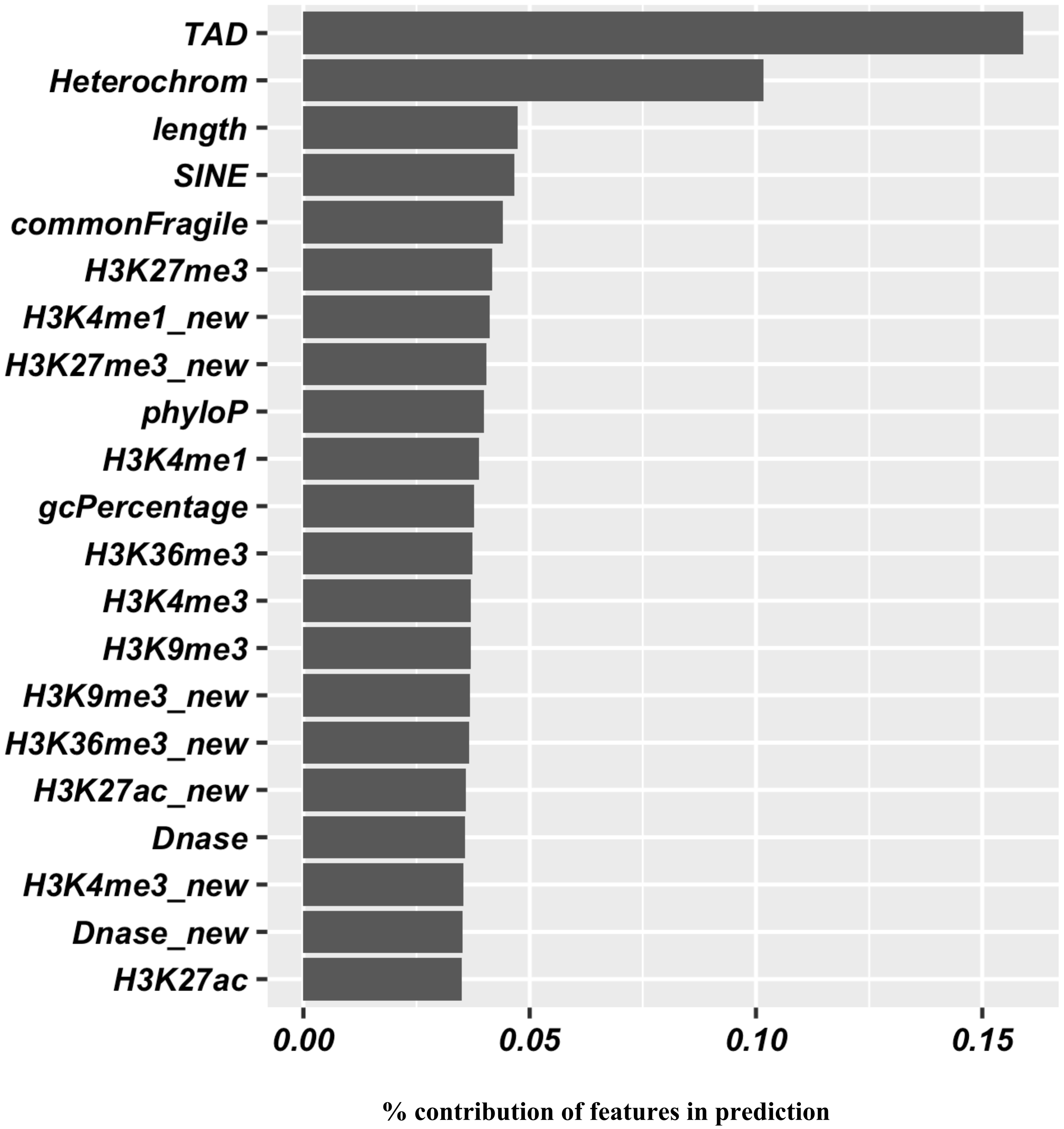

SI FIG S14: Bar plot presenting the percentage contribution for distinct features in the overall predictability of the germline deletion model for the cardiovascular disease cohort. We applied heart tissue-specific TAD definition to build this model.

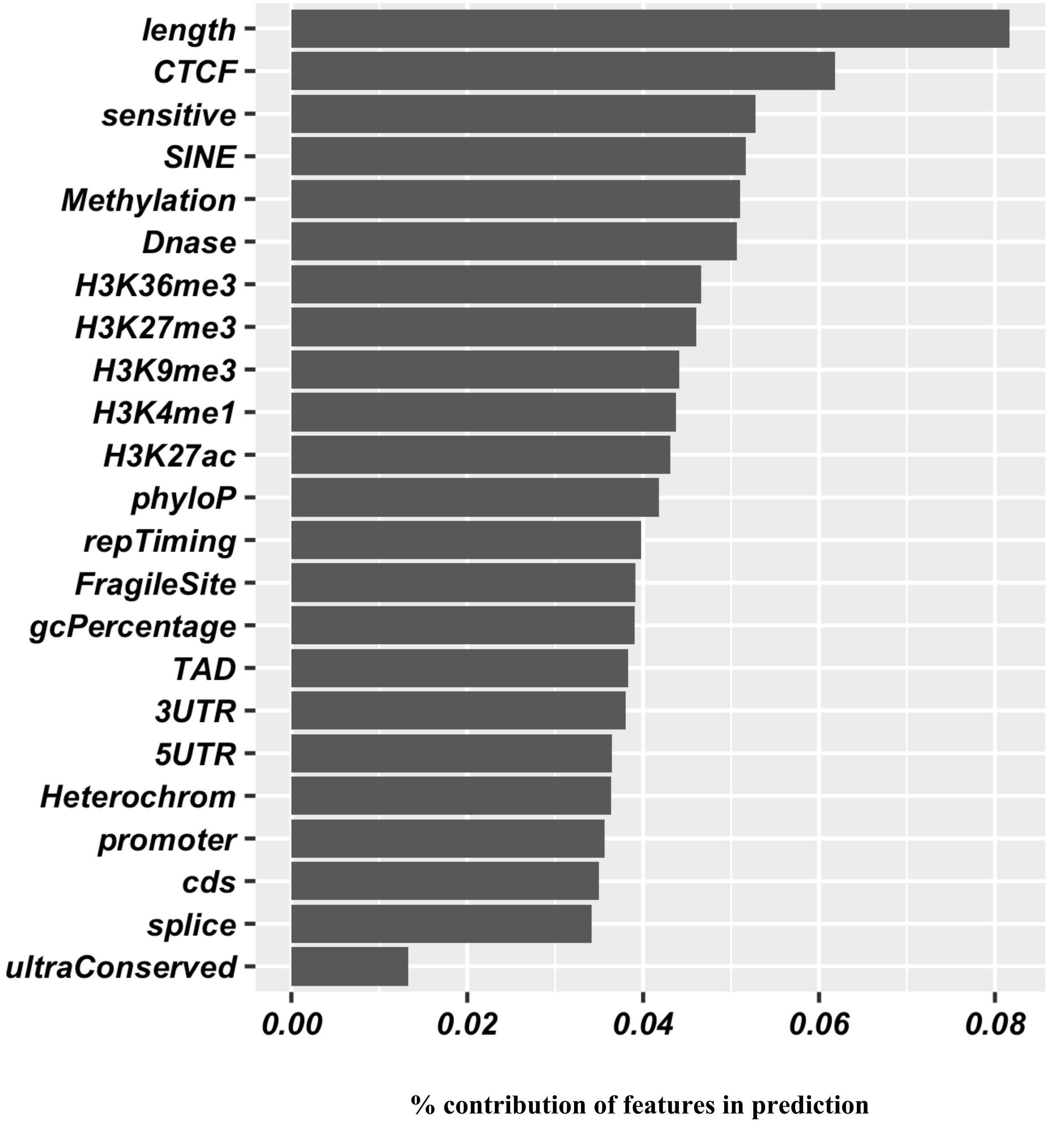

SI FIG S15: Percentage contribution for distinct features in the overall predictability of the germline deletion model for the inflammatory bowel disease cohort.

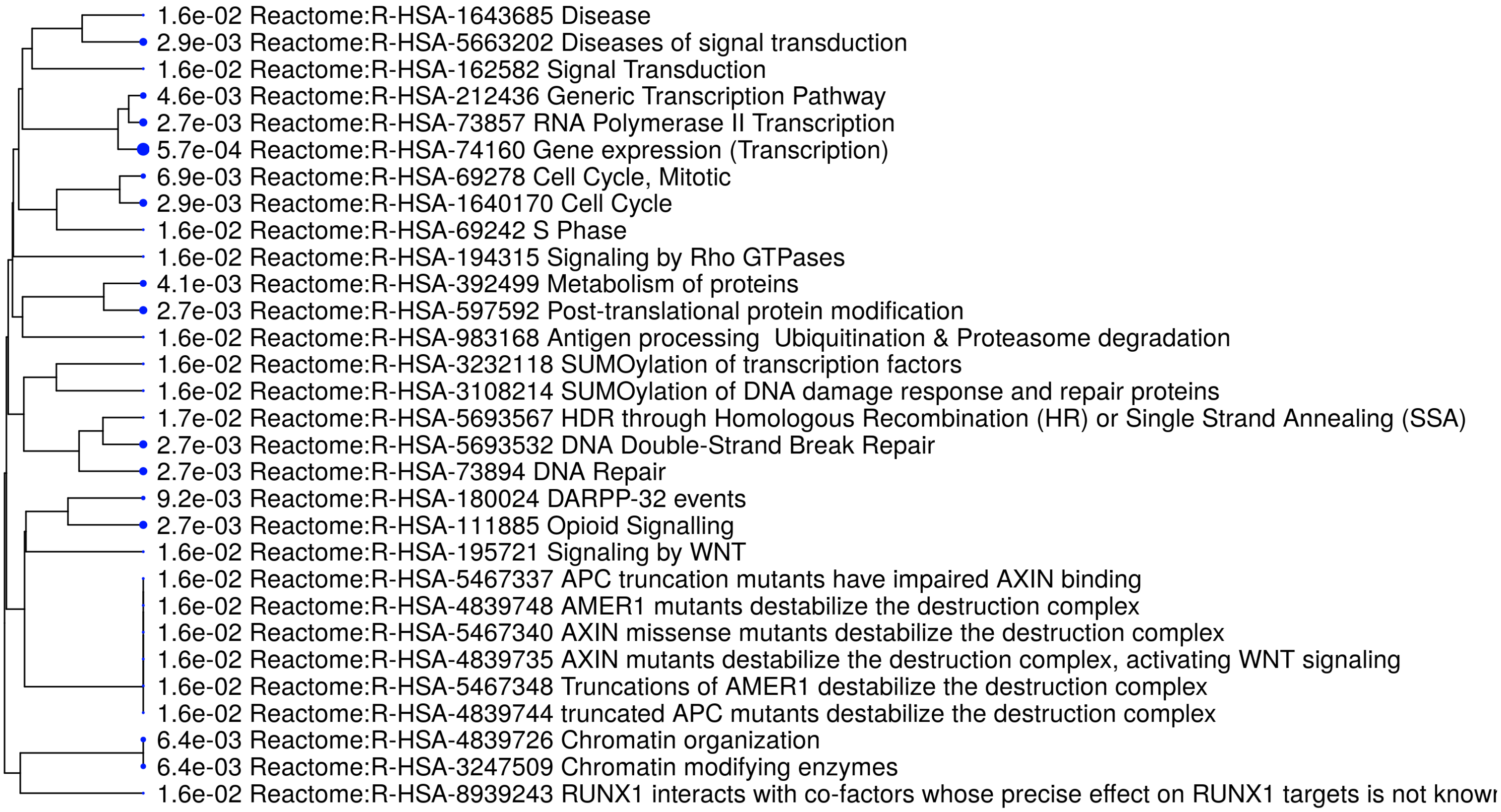

SI FIG S16: Enrichment of genes influence by highly pathogenic somatic deletions in reactome pathways. This analysis was performed on the Pancancer level.

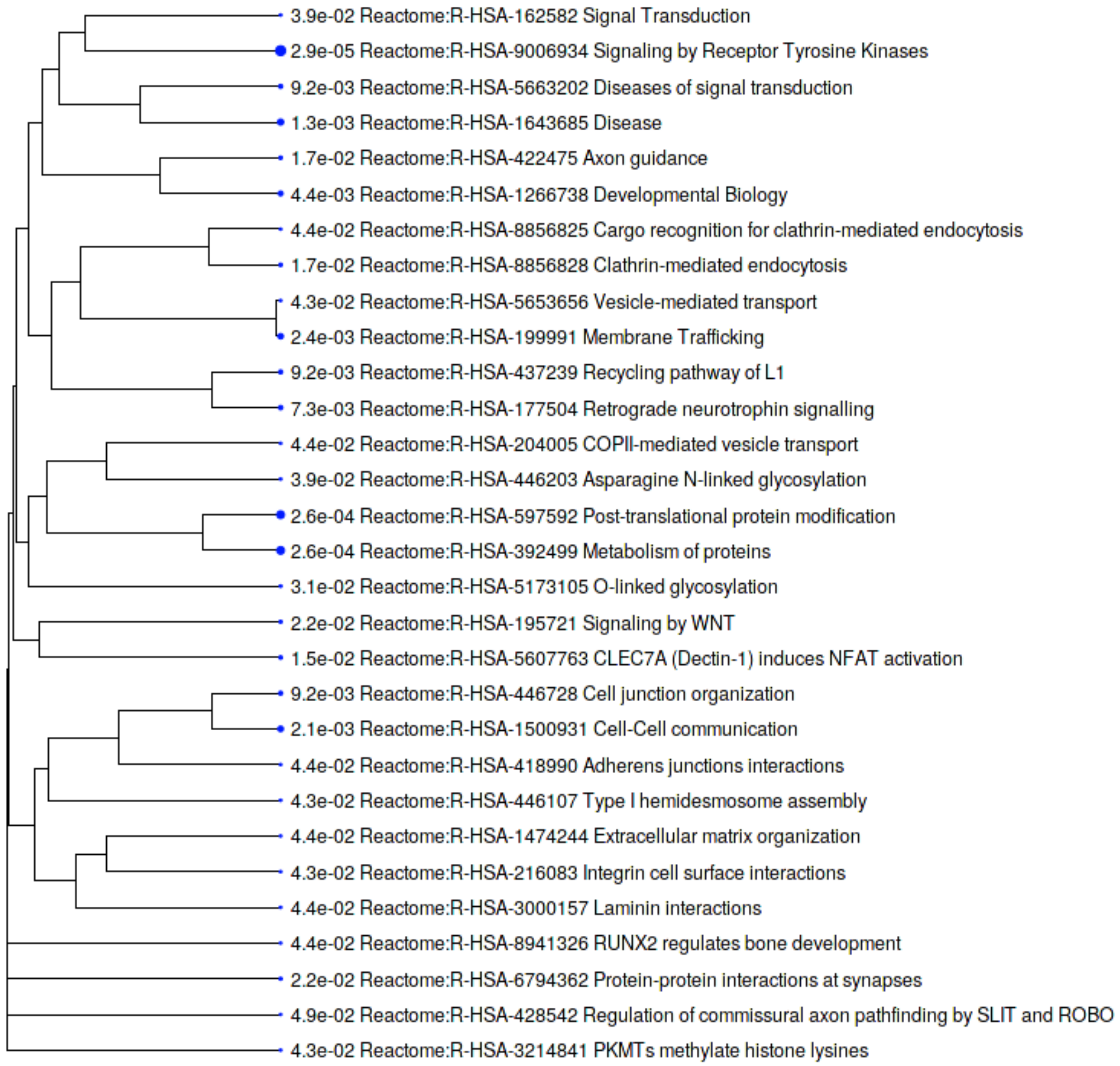

SI FIG S17: Enrichment of genes influence by highly pathogenic somatic duplications in reactome pathways. This analysis was performed on the Pancancer level.

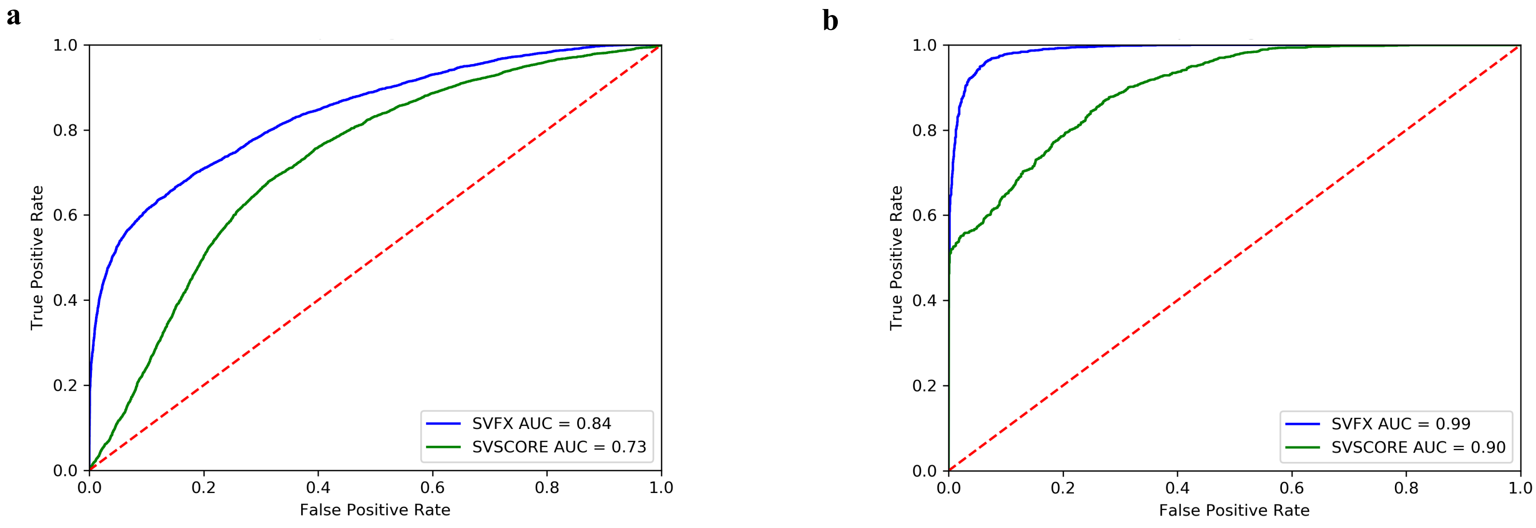

SI FIG S18: *Area under Receiver Operating Characteristics (auROC) based performance comparison between SVFX and SVScore methods.* a) auROC plots for somatic deletions in pan-cancer (aggregated over six cancer cohorts) that were assigned pathogenicity score using SVFX (blue curve) and SVScore (green curve) methods. We excluded these somatic deletions during the training of these models. b) auROC plots for germline deletions in the independent Clinvar datasets that were not used for training either of these models. Here, blue and green curves correspond to the performance of SVFX and SVScore methods, respectively.

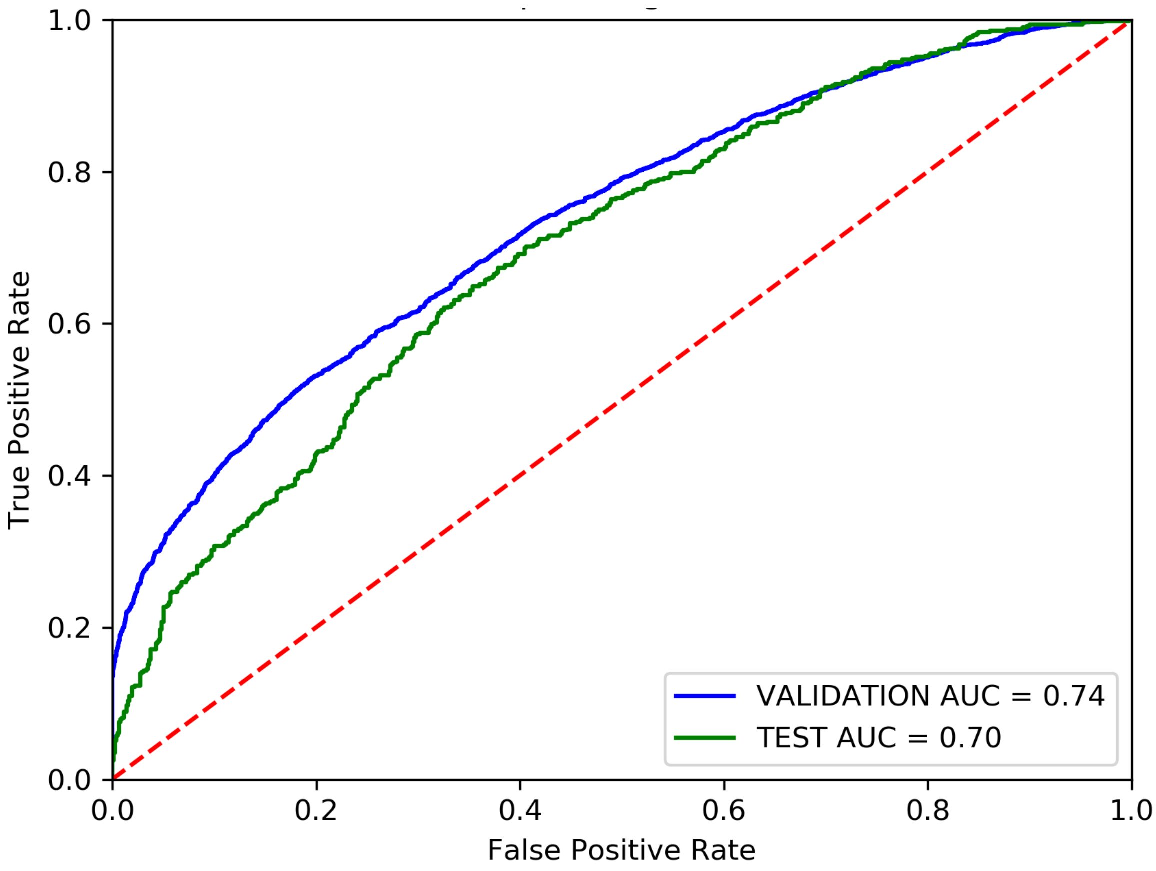

SI FIG S19: *Area under Receiver Operating Characteristics (auROC) for germline deletions in the breast cancer cell line identified by long-read sequencing.* auROC plots for germline deletions in a breast cancer cell line using the 10-fold cross-validation approach and independent testing datasets. These germline deletions were identified using PacBio sequencing data. Blue and green curves represent evaluation using a 10-fold and independent testing dataset, respectively.

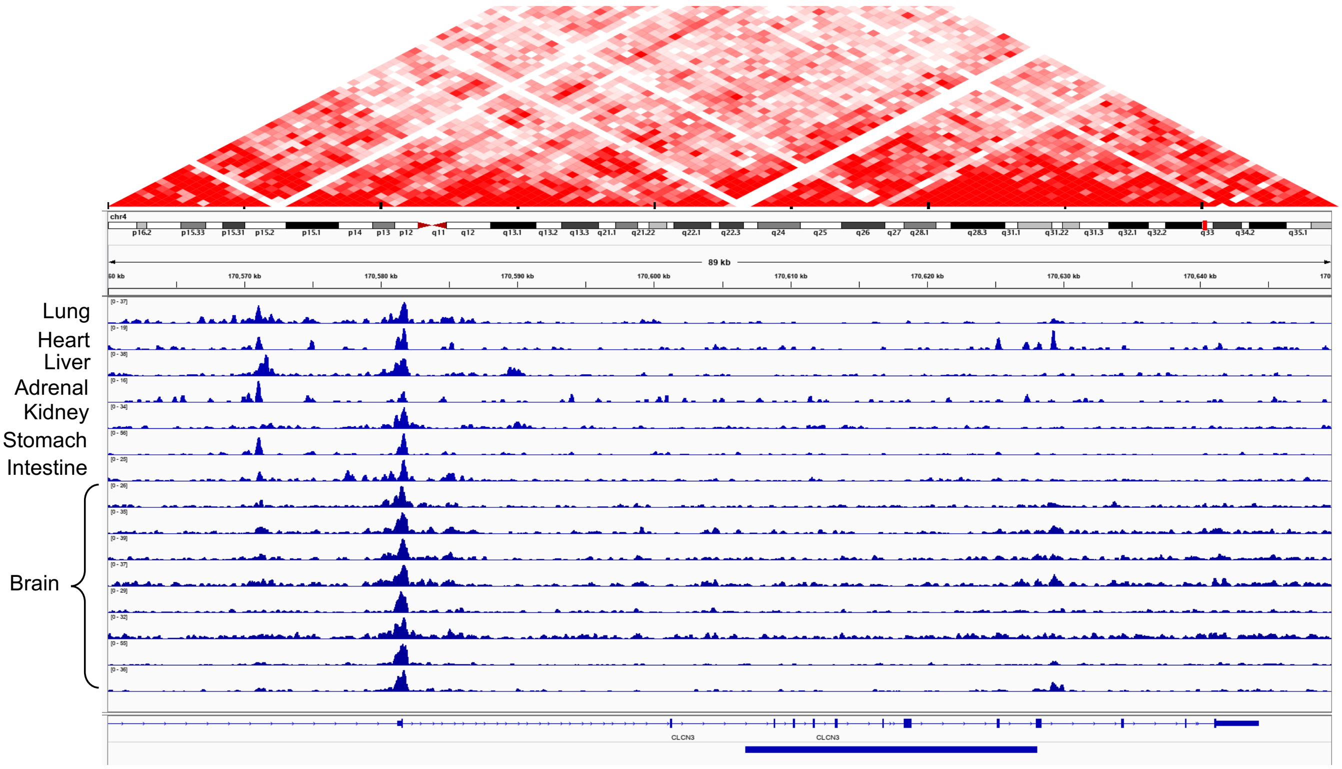

SI FIG S20: *Example of a highly pathogenic deletion in the cardiovascular disease cohort.* This figure presents a highly pathogenic deletion that disrupts the coding region of the *Clcn3* gene. A previous study has shown the potential role of CLcn3 disruption in heart failure. The Hi-C matrix plot shows the TAD boundaries disrupted by this deletion.

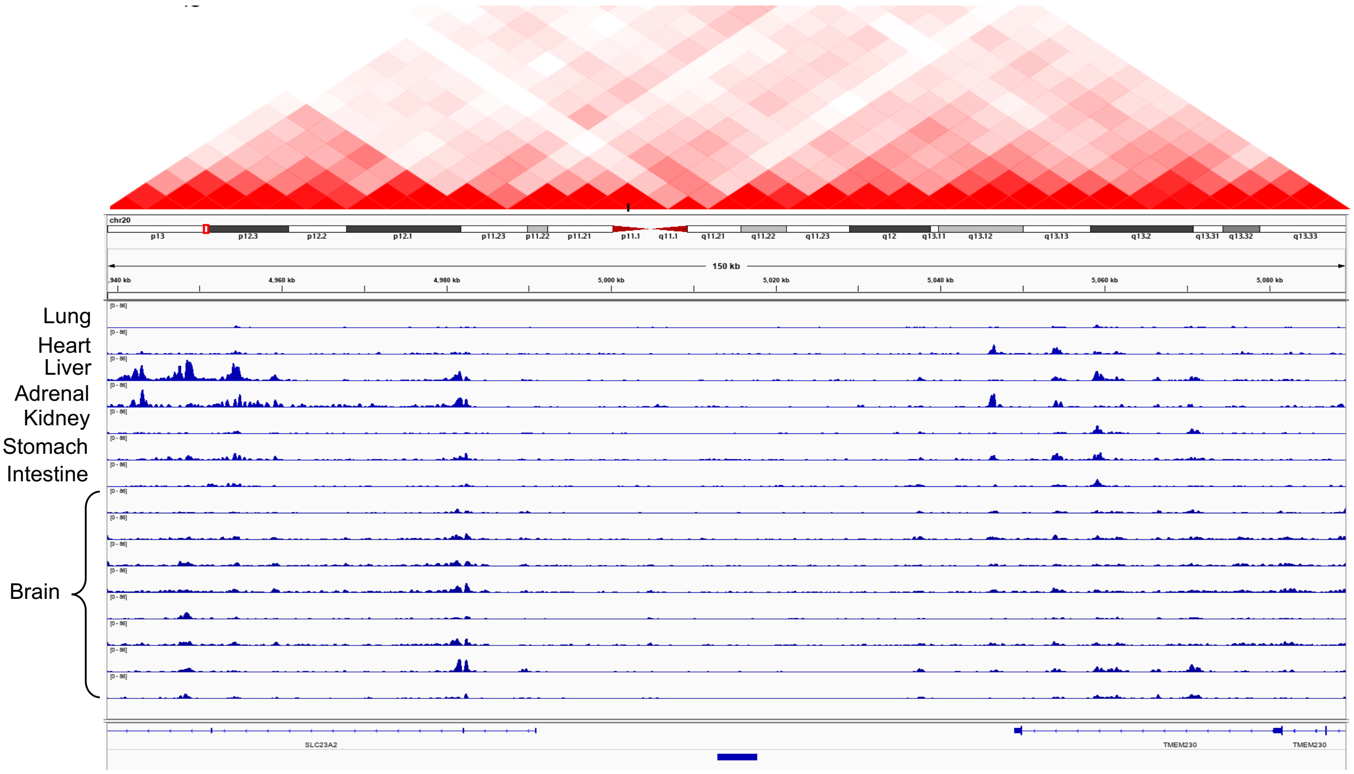

SI FIG S21: *Example of a highly pathogenic deletion in the inflammatory bowel disease cohort.* The above plot highlights a highly pathogenic deletion proximal to the *SLC23A2* gene. This particular deletion overlaps with weak enhancer sites and boundaries of two large TADs and potentially lead to dysregulation of surrounding genes.
