# Supplementary material for: SVFX: a machine learning framework to quantify the pathogenicity of structural variants
Source: Genome Biol. 2020 Nov 9;21:274. doi: 10.1186/s13059-020-02178-x (PMC7650198; doi:10.1186/s13059-020-02178-x)
Supplement: Supplementary file 3 — Additional file 3. Review history. [file 13059_2020_2178_MOESM3_ESM.docx]

**1^st^ round**

**Reviewer 1**

In this manuscript, Kumer et al describe an approach, SVFX, to estimate and score the pathogenicity of structural variation (SV). Previous approaches have been developed to infer the pathogenicity of single nucleotide changes and small insertions and deletions, but very few have focused on the impact of larger SVs. The authors describe a method that employs a random forest-based algorithm to classify the pathogenicity of both germline and somatic SVs by comparing against null distributions of genetic and epigenomic features and then provide several case studies as application.

Overall, this is a significant area of research and such tools would be extremely useful for molecular pathologists and others working in clinical diagnostics. However, there are serious concerns that would need to be addressed prior to publication. While the overall concept and method is strong, the main issue stemming from this work is the lack of validation in external datasets, especially those not involved with cancer. All of the validations performed, except for analysis done in a single CVD cohort, are performed on the same cancer datasets for which SVs are traditionally an abundant hallmark and typically larger than in other diseases. Furthermore, there are no comparisons provided to other similar approaches (e.g. SVScore) to show what level of advancement SVFX provides. Finally, the manuscript is difficult to read and would benefit from additional editing and explicit details provided as to what specifically was done. Specific comments are below.

Major comments

1. A primary argument within the manuscript is that the integration of different genetic/genomic features improves the ability to predict the pathogenicity of a given variant, assuming that pathogenic variants differ at these features when compared to benign variants. However, this is never explicitly proven. It is necessary to show whether or not the data-driven approach SVFX takes is more accurate than the cited SVScore (Ganel et al., Bioinformatics, 2017) which computes their pathogenicity measure through individual base-pair scoring. Comparisons should be done both with the same datasets that each algorithm is trained on as well as datasets that neither is trained on.

2. The authors use the 1KG variants as 'benign', but it is not clear if they use all reported SVs (including MEIs) or just a subset with some common allele frequency cut-off (see methods comment below). The first paragraph of the Results section states that they set "number of germline SVs in the disease set is same(sic) as the common SVs (global allele frequency >0.5%), but it is not clear if this is what was used for the full benign set and nothing is provided in the Methods section to answer this. The authors need to be more explicit as to what specifically was used to train their model.

3. A significant portion of the SV field is currently focused on using genome technologies beyond short-reads, as short-reads are only able to resolve a fraction of detectable SVs (Chaisson et al., Nature Communications, 2019). This is shortly touched upon in the discussion portion but begs the following question: is SFVX extendable to long-read datasets? Specifically, would SFVX perform strongly in detecting cancer SVs sequenced with long-read technologies (Nattestad et al., Genome Research, 2018) when compared to a potential "benign or null" set (Audano et al., Cell, 2019)?

4. There are no mentions of insertions, inversions, or translocations until the discussion section. While the exclusion of these SV classes make sense from a technical standpoint due to the difficulty in resolving these SV types, it should be explicitly addressed at the beginning of the manuscript. Ideally, these SV types are included in a re-analysis. It is difficult to say that a given method excels at detecting disease SVs if SV subtypes are being left out. Additional ROC curves for at least the most common types of SVs (mentioned above) should be incorporated with Figure 2.

5. The datasets used for classification and subsequent AUC quantification in Figure 2 are all cancer datasets. For the single external analysis (CVD), the overall auROC and auPRC plots are lower than that for the training data. Thus, a more thorough application to other potential SV datasets is required to rule out overfitting given that these datasets were specifically chosen due to the availability of tissue-specific epigenomic markers. How will SFVX perform when it is generalized to datasets for which these resources are not available? Additionally, how would SFVX perform when SVs are less of a functional hallmark than they are in cancer, such as in ASD cohorts, where SVs are suggested to be more in line with the common disease, common variant hypothesis?

6. The first paragraph of page 11 refers to figure S10 showing high relative contribution of TAD overlap to the predictability of germline SVs. The TADs used in this model are derived from Dixon et al., Nature, 2015, which performed Hi-C in differentiating embryonic stem cells. However, TADs are dynamic and cell-type + timepoint specific. If the annotations are replaced with TADs from a different dataset derived from a different cell type, will they still contribute strongly and how much does this affect the prediction of germline variants?

7. The methods section is missing many details required for the reproducibility of the stated work. For example, the authors state that 'random structural variant coordinates were generated across the genome', however it is not clear if this is the 'full' genome or the 'accessible' genome; that is, do the authors enable the placement of random SVs into sub-telomeric or peri-centromeric regions, and what impact would this have on ascertainment from short-read technologies? In general, the methods section is very qualitative/descriptive and does not provide enough specific details for reproduction.

Minor comments

*	While the title and much of the manuscript refer to "pathogenic" variants, the algorithm described classifies variants present in disease cohorts, not truly "pathogenic" variants of which should only comprise a small subset of disease variants. The manuscript itself mentions this twice, once in the results and once in the methods, but the classifier is more of a "disease" classifier than a pathogenicity classifier and thus statements about deleteriousness should be adjusted lest they remain misleading for the reader.

*	The case studies should ideally include a disease that is not cancer-based, to see if such downstream analyses are useful for those working on other diseases.

*	Actual numbers of SVs per cohort and correctly classified SVs should be provided.

*	There is mention of a recurrence-based analysis on page 9, line 52 but nothing that points toward where these scores are available.

*	For reproducibility, table S1 should explicitly reference the source for each of these annotations given that many different lists of annotations have been developed. While broad information is provided on GitHub, specific URLs would be extremely helpful.

This manuscript requires rewording as there are grammatical errors throughout. Below, I have pointed out some easy changes.

Page 7, line 30 - I am not sure that "functional annotation" contributes to the distribution of SVs. A potential rewording might be "regulatory role" or functional embedding

Page 8, line 3 - "heterochromatin regions" to "heterochromatic regions"

Page 8, line 44 - "using random forest algorithm" to "using random forests

Page 9, line 13 - "play the role of cancer drives, whereas low score SVs were likely to be passenger..." to "play the role of cancer drivers, whereas low score SVs would likely be passenger…"

Page 13, line 58 - "and thus influence their" to "and thus influences their"

Page 14, line 22 - "of the regulatory element effected" to "of the regulatory element affected"

(methods) page 17, line 46 - "as discusses earlier" to "as discussed earlier"

Figures should carefully be re-ordered according to the manuscript., e.g. figure S8 is mentioned in the manuscript before Fig S3.

**Reviewer 2**

In the manuscript "SVFX: a machine-learning framework to quantify the pathogenicity of structural variants" Kumar and colleagues build a model to predict pathogenicity of structural variants on somatic and germline SVs from disease vs. healthy cohorts. The study is well motivated, describes a novel method to detect putative pathogenic SVs and the reader is nicely introduced into the urgency of such a tool. However, the manuscript lacks crucial information and needs to be revised.

Major:

1) Existing methods (like SVScore or AnnotSV) are not compared.

2) Performance of the machine-learning tool seems rather high on all tested datasets. Further, SV-length (or correlates) were identified as top model features. However, a comparison of length distributions between the labeled training data sets is missing. More generally, ascertainment biases in SVs training data in the 1000G dataset vs the cancer disease cohorts are not addressed. It would be interesting to see model performance in a length matched training data set or after trying to remove SV length and correlates (i.e. using only proportion of bases with a certain annotation as features) to get a feeling of the effect size. While length is an obvious predictor of SV pathogenicity, technical differences between the labeled training data sets need to be excluded. This cannot be addressed by hold-out or cross-fold validation. Thus, model performance should be evaluated on an independent data set, e.g. ClinVar (pathogenic vs. benign/common) and/or gnomAD SV calls (e.g. rare vs. common), to substantiate that the model did not just learn technical differences between two labeled sets.

3) I would also like to clearly state that a Z-score normalization (even though claimed otherwise by the authors) does not correct for such ascertainment biases. If done over all classes, it is only a feature scaling method. If done separately for each class of the training data set, differences in variance would remain predictive and predictions of new data points could not easily be performed as their scaling would be unclear (as their class/ascertainment would need to be matched).

4) Relating to the last sentence of point 2, results are being validated using conservation score enrichment. However, other datasets should be available like the performance on ClinVar SVs, known pathogenic cancer SVs or correlates with allele frequency. This seems especially relevant to substantiate the claim, that the developed method can be applied in cancer as well as other diseases.

5) Generally, the manuscript misses in depth information about properties of model training. Besides the mentioned length distributions or unclear information about applied transformations in a prediction setting, very basic numbers like the number of training examples, information about data balancing, or model hyperparameters are missing. Steps like the introduced 10bp binning and averaging come without any motivation (computational considerations?).

Minor:

- The paper is well written but at times repetitive, i.e. restating already given information without going into additional detail.

- Considering that the authors implicate pathogenic, or cancer SVs in this study it would be relevant to give access to these results (aggregated, i.e. without releasing an association with patients identifiers).

**Authors’ response**

Reviewer 1:

While the overall concept and method is strong, the main issue stemming from this work is the lack of validation in external datasets, especially those not involved with cancer. All of the validations performed, except for analysis done in a single CVD cohort, are performed on the same cancer datasets for which SVs are traditionally an abundant hallmark and typically larger than in other diseases. Furthermore, there are no comparisons provided to other similar approaches (e.g. SVScore) to show what level of advancement SVFX provides. Finally, the manuscript is difficult to read and would benefit from additional editing and explicit details provided as to what specifically was done. Specific comments are below.

We thank the reviewer for appreciating the strength of our work. Based on their feedback, now we have updated our manuscript to include assessment of our method in additional disease cohorts and comparison with the SVScore method. We have also updated the methods section to provide more explicit details of our approach.

A primary argument within the manuscript is that the integration of different genetic/genomic features improves the ability to predict the pathogenicity of a given variant, assuming that pathogenic variants differ at these features when compared to benign variants. However, this is never explicitly proven. It is necessary to show whether or not the data-driven approach SVFX takes is more accurate than the cited SVScore (Ganel et al., Bioinformatics, 2017) which computes their pathogenicity measure through individual base-pair scoring. Comparisons should be done both with the same datasets that each algorithm is trained on as well as datasets that neither is trained on.

We thank the reviewer for this suggestion. We have performed comparison of our method with SVScore algorithm using independent cancer dataset that was not used for training. Additionally, we also perform such comparison with Clinvar SVs that were not employed for training. Overall, our method performs significantly better (with higher AUC score) than SVScore approach in both of these independent datasets.

Change to manuscript: *We also compared our data-driven approach with the prior SVScore method [14] that assigns deleteriousness scores to SVs by applying a pre-computed base-pair score (measured for assessing the impact of single-nucleotide changes). We found that our SVFX method performed significantly better than SVScore in identifying pathogenic SVs in both somatic and germline contexts. For instance, in our independent testing dataset for various cancer cohorts, our somatic deletion model had better precision and recall (average auROC value of 0.84) compared to SVScore (an average auROC of 0.73) across multiple cancer cohorts (supplement Fig S18a). Similarly, for the germline ClinVar[16] SV model, our approach showed significantly better performance (auROC of 0.99) compared to the SVScore method (auROC value of 0.9) in the independent testing dataset (supplement Fig S18b). These observations further highlight the efficacy of our approach for detecting pathogenic SVs.*

The authors use the 1KG variants as 'benign', but it is not clear if they use all reported SVs (including MEIs) or just a subset with some common allele frequency cut-off (see methods comment below). The first paragraph of the Results section states that they set "number of germline SVs in the disease set is same(sic) as the common SVs (global allele frequency >0.5%), but it is not clear if this is what was used for the full benign set and nothing is provided in the Methods section to answer this. The authors need to be more explicit as to what specifically was used to train their model.

We apologize for this confusion. For distinct SV categories (deletions and duplications), we build different models by utilizing SVs belonging to those individual categories, thus MEIs were not used in training our models. In case of germline models, for training purpose we sub select common SVs from 1KG dataset to match the number of disease germline SVs in our cancer cohort. We have now updated our method section to clarify this further.

Change to manuscript: *Furthermore, to assemble these disease and benign SVs datasets, we also considered the underlying SV types. For instance, our somatic deletion models include only deletions (with length > 50 bp) that are present in the cancer cohort and 1KG SV datasets. Similarly, our duplication models are comprised of duplications that are present in the disease and corresponding control/benign cohorts. Overall, the number of deletions was significantly*

A significant portion of the SV field is currently focused on using genome technologies beyond short-reads, as shortreads are only able to resolve a fraction of detectable SVs (Chaisson et al., Nature Communications, 2019). This is shortly touched upon in the discussion portion but begs the following question: is SFVX extendable to long-read datasets? Specifically, would SFVX perform strongly in detecting cancer SVs sequenced with long-read technologies (Nattestad et al., Genome Research, 2018) when compared to a potential "benign or null" set (Audano et al., Cell, 2019)?

We thank the reviewer for this great suggestion. We trained and assessed the performance our method by utilizing deletions from long read (using the datasets from Nattestad et al. and Audano et al.) as mentioned by the reviewer. Overall, our long-read based trained model had similar but slightly lower accuracy (auc= 0.74) for the breast cancer sample compared to our original short-read based breast cancer cohort model (auc =0.78). This slight lower in accuracy can be attributed to the smaller sample size in the long-read based disease and benign cohorts.

Change to manuscript: *In recent years, there has been increasing interest in characterizing accurate SV maps for human genomes using long-read sequencing platforms[33]. Our method is highly flexible and can be easily applied to SVs identified using long-read sequencing data. Despite the relative superior performance of long-read sequencing platforms in identifying SVs, we currently lack a large-scale SV dataset based on these approaches. Thus, as a proof of principle, we applied our method on deletions identified using long-read sequencing data for a breast cancer cell line[34] and "benign deletions" identified in healthy individuals[35]. We note that the number of samples in these datasets is relatively limited. However, we observed similar performance for our longread germline deletion model compared to those using deletions based on short-read sequencing data (supplement Fig S19). Overall, for the ten-fold cross-validation (auROC value of 0.74) and the hold-out testing datasets (auROC value of 0.7), our model showed good performance.*

There are no mentions of insertions, inversions, or translocations until the discussion section. While the exclusion of these SV classes make sense from a technical standpoint due to the difficulty in resolving these SV types, it should be explicitly addressed at the beginning of the manuscript. Ideally, these SV types are included in a re-analysis. It is difficult to say that a given method excels at detecting disease SVs if SV subtypes are being left out. Additional ROC curves for at least the most common types of SVs (mentioned above) should be incorporated with Figure 2.

We thank the reviewer for this suggestion. Unfortunately, as the reviewer points out due to technical challenges associated with shortread based sequencing platform, we currently lack sufficient amount of high-quality inversions, insertions and translocations in our disease and benign cohorts. Thus, training an accurate model becomes very challenging. We envision that long-read sequencing platform will address this challenge in coming years. We discuss this limitation in the updated manuscript.

Change to manuscript: *Additionally, our current framework primarily focuses on identifying pathogenic deletions and duplications. However, it can be readily extended to detect pathogenic inversions and translocations in these diseases. We note that, currently, there is a lack of high-quality inversions and translocations in public databases, including 1KG, gnomAD, and ClinVar SV databases, limiting the applicability of our approach toward distinguishing all disease-associated SVs from benign ones. However, the rapid rise in long-read base sequencing platforms and their inherent ability to resolve inversions, translocations, and complex SVs will facilitate the generation of comprehensive SV resources. In the future, we can employ such extensive resources to train somatic and germline models to detect highly pathogenic inversions and translocations from benign ones.*

The datasets used for classification and subsequent AUC quantification in Figure 2 are all cancer datasets. For the single external analysis (CVD), the overall auROC and auPRC plots are lower than that for the training data. Thus, a more thorough application to other potential SV datasets is required to rule out overfitting given that these datasets were specifically chosen due to the availability of tissue-specific epigenomic markers. How will SFVX perform when it is generalized to datasets for which these resources are not available? Additionally, how would SFVX perform when SVs are less of a functional hallmark than they are in cancer, such as in ASD cohorts, where SVs are suggested to be more in line with the common disease, common variant hypothesis?

We appreciate the reviewer’s concern related to lack of tissue-specific epigenomic markers. We now test our method on additional common and rare disease-associated SVs. First, we tested a generic germline model for Clinvar SV dataset, where we didn’t employ any tissuespecific epigenomic data. This generic model (based on GM12878 cell type) performed similar to other tissue-specific germline models in different disease setting. Furthermore, we also tested our model’s performance in IBD disease cohort, where SVs role fall in line with common disease, common variant hypothesis. Our IBD model performed similar to cardiovascular disease cohort, which we evaluated earlier.

Change to manuscript: *We note that our current framework is highly flexible and can be easily applied to identify pathogenic germline SVs for various common and rare diseases. Thus, we applied our approach to detect pathogenic germline SVs in CVD and IBD cohorts (included in the GSP[29]). Furthermore, we identified putative pathogenic SVs in the ClinVar SV dataset, which is enriched with rare disease-associated SVs.*

*…………………………..*

*Furthermore, we utilized large deletions in the recently released gnomAD-SV dataset as a control for the germline IBD model. Overall, due to the lower number of deletions in our training and testing datasets, we observed a slight decrease in the performance of our IBD-specific germline model, which had mean auROC and auPR values of 0.73 and 0.71, respectively, based on the ten-fold cross-validation approach (Fig 2e & supplement Fig S11). Our model's performance on the testing dataset for this cohort was comparable, with, a mean auROC and auPR value of 0.72 (Fig 2f & supplement Fig S11). Finally, we utilized known pathogenic and benign SVs, as reported in the ClinVar SV database, to build this model. We note that ClinVar database include SVs from a wide range of diseases. Thus, we utilized functional & epigenomics data from a generic cell line (GM12878) to train our ClinVar germline models. Compared with data for common diseases such as CVD and IBD, the ClinVar SV database is highly enriched with rare and likely deleterious SVs. Thus, our model achieved near-perfect accuracy and recall (auROC and auPR values of 0.99) while distinguishing pathogenic ClinVar deletions and duplications from benign SVs (Fig 2e-f & supplement Fig S12).*

The first paragraph of page 11 refers to figure S10 showing high relative contribution of TAD overlap to the predictability of germline SVs. The TADs used in this model are derived from Dixon et al., Nature, 2015, which performed Hi-C in differentiating embryonic stem cells. However, TADs are dynamic and cell-type + timepoint specific. If the annotations are replaced with TADs from a different dataset derived from a different cell type, will they still contribute strongly and how much does this affect the prediction of germline variants?

Based on reviewer’s suggestion, we replaced TAD annotation to a tissue-specific one. In this modified model, the updated TAD annotation contributed strongly similar to our previous germline SVFX model.

Change to manuscript: *We note that TADs are highly conserved across multiple tissues and cell types[30]. Thus, upon replacement of the TAD definition (based on a different cell type) in our original CVD model, we observed a similar contribution of TAD annotation to the predictability of the modified model (supplement Fig S14).*

The methods section is missing many details required for the reproducibility of the stated work. For example, the authors state that 'random structural variant coordinates were generated across the genome', however it is not clear if this is the 'full' genome or the 'accessible' genome; that is, do the authors enable the placement of random SVs into sub-telomeric or peri-centromeric regions, and what impact would this have on ascertainment from short-read technologies? In general, the methods section is very qualitative/descriptive and does not provide enough specific details for reproduction.

We have now updated the method section of our manuscript to provide necessary specific details. Briefly, we restrict randomization of a given SV on the same chromosome. For our analyses, we allowed randomization across the entire chromosome. However, we also allow users to place random SVs only in ‘accessible regions’ of the genome.

While the title and much of the manuscript refer to "pathogenic" variants, the algorithm described classifies variants present in disease cohorts, not truly "pathogenic" variants of which should only comprise a small subset of disease variants. The manuscript itself mentions this twice, once in the results and once in the methods, but the classifier is more of a "disease" classifier than a pathogenicity classifier and thus statements about deleteriousness should be adjusted lest they remain misleading for the reader.

We thank the reviewer for the suggestion. We have updated the discussion section to further clarify the interpretation of our “pathogenicity score” terminology to avoid this confusion.

Change to manuscript: *We note that our SV pathogenicity score quantify the probability of given SVs belonging to a particular disease cohort compared to being present in a healthy population. Thus, one could interpret our models as disease classifiers rather than frameworks that explicitly quantify the endophenotypic effect of a given SV.*

The case studies should ideally include a disease that is not cancer-based, to see if such downstream analyses are useful for those working on other diseases.

We now provide additional case studies for the cardiovascular and inflammatory bowel disease cohorts in the updated manuscript.

Change to manuscript: *Finally, we present a few examples of highly pathogenic germline deletions in the CVD and IBD cohorts. For instance, one of the predicted highly pathogenic deletions in our CVD cohort overlaps with the coding region of the Clcn3 gene, which encodes the voltage-gated chloride channel protein CLC-3 (supplement Fig S20). A previous study showed that the deletion of Clcn3 potentially affects the ion channel involved in cell volume homeostasis, which likely drives the development of myocardial hypertrophy and heart failure[43]. Similarly, we identified a putative pathogenic deletion in the IBD cohort that is proximal to the promoters of the SLC23A2 gene (supplement Fig S21), which has essential roles in vitamin C metabolism that are directly associated with the manifestation of IBD[44]. This deletion overlaps with weak enhancer sites and boundaries of two large TADs, which can be potentially deleterious to the proper regulation of the surrounding genes. These examples example further highlight the ability of our method to detect biologically relevant pathogenic SVs in common disease cohorts.*

Actual numbers of SVs per cohort and correctly classified SVs should be provided.

In the updated manuscript, we now report number of SVs per cohort in different disease contexts.

Change to manuscript: *For instance, our somatic deletion models include only deletions (with length > 50 bp) that are present in the cancer cohort and 1KG SV datasets. Similarly, our duplication models are comprised of duplications that are present in the disease and corresponding control/benign cohorts. Overall, the number of deletions was significantly higher than duplications in our disease and corresponding benign datasets (supplement data table 7).*

There is mention of a recurrence-based analysis on page 9, line 52 but nothing that points toward where these scores are available.

We apologize for the confusion. We now provide relevant details related to this analysis along with scores for these driver SVs in our updated manuscript and supplement section.

Change to manuscript: *Furthermore, we quantified the pathogenic score for large deletions and duplications that are predicted to be driver events on a pan-cancer level using a recurrence-based analysis[28]. As expected, our workflow assigned a high pathogenic score (average score greater than 0.85 across different somatic models) to each putative driver event (supplement data table2).*

For reproducibility, table S1 should explicitly reference the source for each of these annotations given that many different lists of annotations have been developed. While broad information is provided on GitHub, specific URLs would be extremely helpful.

We thank the reviewer for this suggestion. We now provide specific URLs for different annotations utilized in our model generation process.

This manuscript requires rewording as there are grammatical errors throughout. Below, I have pointed out some easy changes.

Page 7, line 30 - I am not sure that "functional annotation" contributes to the distribution of SVs. A potential rewording might be "regulatory role" or functional embedding

Page 8, line 3 - "heterochromatin regions" to "heterochromatic regions"

Page 8, line 44 - "using random forest algorithm" to "using random forests

Page 9, line 13 - "play the role of cancer drives, whereas low score SVs were likely to be passenger..." to "play the role of cancer drivers, whereas low score SVs would likely be passenger…"

Page 13, line 58 - "and thus influence their" to "and thus influences their"

Page 14, line 22 - "of the regulatory element effected" to "of the regulatory element affected" (methods) page 17, line 46 - "as discusses earlier" to "as discussed earlier"

We have updated the manuscript to address these grammatical mistakes in the manuscript.

Figures should carefully be re-ordered according to the manuscript., e.g. figure S8 is mentioned in the manuscript before Fig S3.

Based on the reviewer’s suggestion, we have reordered our supplemental figures.

Reviewer 2:

In the manuscript "SVFX: a machine-learning framework to quantify the pathogenicity of structural variants" Kumar and colleagues build a model to predict pathogenicity of structural variants on somatic and germline SVs from disease vs. healthy cohorts. The study is well motivated, describes a novel method to detect putative pathogenic SVs and the reader is nicely introduced into the urgency of such a tool. However, the manuscript lacks crucial information and needs to be revised.

We thank the reviewer for the positive comment and highlighting the novelty of our work. We have updated the manuscript to address the issue of missing details particularly in the method section.

Existing methods (like SVScore or AnnotSV) are not compared.

We thank the reviewer for this suggestion. We have performed comparison of our method with SV score and report it in the updated manuscript.

Change to manuscript: *We found that our SVFX method performed significantly better than SVScore in identifying pathogenic SVs in both somatic and germline contexts. For instance, in our independent testing dataset for various cancer cohorts, our somatic deletion model had better precision and recall (average auROC value of 0.84) compared to SVScore (an average auROC of 0.73) across multiple cancer cohorts (supplement Fig S18a). Similarly, for the germline ClinVar[16] SV model, our approach showed significantly better performance (auROC of 0.99) compared to the SVScore method (auROC value of 0.9) in the independent testing dataset (supplement Fig S18b). These observations further highlight the efficacy of our approach for detecting pathogenic SVs.*

Performance of the machine-learning tool seems rather high on all tested datasets. Further, SV-length (or correlates) were identified as top model features. However, a comparison of length distributions between the labeled training data sets is missing. More generally, ascertainment biases in SVs training data in the 1000G dataset vs the cancer disease cohorts are not addressed. It would be interesting to see model performance in a length matched training data set or after trying to remove SV length and correlates (i.e. using only proportion of bases with a certain annotation as features) to get a feeling of the effect size.

We thank the reviewer for pointing out a potential issue related to ascertainment bias caused by the length distribution imbalance of SVs in the training data. Based on reviewer’s suggestion, we assessed the performance of modified models by removing SV length and other correlates. Removal of length as feature had a minimal effect on the accuracy of our original model.

Change to manuscript: *We note that, to a certain extent, the use of Z-score-normalized feature matrices helped us avoid implicit bias in our somatic models due to differences in the length distribution of cancer-associated and 1KG SVs. However, we also included the SV length as an explicit feature in our final somatic models to assign high pathogenicity scores to extremely large SVs, such as chromosomal arm deletions and duplications. The inclusion of SV length as an explicit feature can potentially bias the predictive performance of our model. Thus, we quantified the predictability differences for the original models when the SV length was removed as a feature. Overall, we found that the AUC value was 3% lower, on average, for truncated somatic models that lacked SV length as a specific feature (supplement Fig 4).*

While length is an obvious predictor of SV pathogenicity, technical differences between the labeled training data sets need to be excluded. This cannot be addressed by hold-out or cross-fold validation. Thus, model performance should be evaluated on an independent data set, e.g. ClinVar (pathogenic vs. benign/common) and/or gnomAD SV calls (e.g. rare vs. common), to substantiate that the model did not just learn technical differences between two labeled sets.

Based on the reviewer’s suggestion, we applied our approach on Clinvar SV calls (pathogenic vs. benign/common). Overall, our Clinvar model performed very well (with mean auc of 0.99). We also note that our somatic model assigned high pathogenic score to previously identified drivers.

I would also like to clearly state that a Z-score normalization (even though claimed otherwise by the authors) does not correct for such ascertainment biases. If done over all classes, it is only a feature scaling method. If done separately for each class of the training data set, differences in variance would remain predictive and predictions of new data points could not easily be performed as their scaling would be unclear (as their class/ascertainment would need to be matched).

We apologize for the confusion. However, the Z-score normalization was not performed within or between SV classes. For each SV (disease or benign), we generated 1000 random instances of SVs from the same chromosome where the length of random SV was the same as the original SV. We applied Z-score normalization using these randomized and the original SVs. As each SV feature was scaled relative to features of randomized SVs of the same length, we believe that this normalization approach should significantly weaken or remove length bias in the dataset.

Change to manuscript: *As expected, we observed significant disparity in the length distribution for SVs in our disease cohort and control datasets, especially in the somatic context (supplement Fig S1). These length differences are likely to influence implicitly various features necessary to train our machine learning model. Thus, to avoid the effect of such length bias on our models, we generated random SV coordinates for both disease and benign datasets. For each original SV (deletion or duplication) in the disease and benign set, we uniformly sampled one thousand randomized SV instances. Each of the randomized SVs had the same length and occupied the same chromosome as the original SV.*

*..……………………….*

*After extracting the relevant features for both disease-associated and benign SVs, we normalized the original feature matrix. The feature normalization was essential to avoid any implicit feature bias due to length distribution differences observed between disease and benign SV datasets. For a given feature, we computed the feature value of each original SV and the corresponding values for the thousand randomly shuffled SV instances of that particular SVs. Subsequently, we applied a Z-Score transformation to perform the normalization for disease-associated and benign SV dataset.*

Relating to the last sentence of point 2, results are being validated using conservation score enrichment. However, other datasets should be available like the performance on ClinVar SVs, known pathogenic cancer SVs or correlates with allele frequency. This seems especially relevant to substantiate the claim, that the developed method can be applied in cancer as well as other diseases.

We thank the reviewer for these suggestions. Previously, we showed that our approach assigns high score to previously characterized driver cancer SVs. In the updated manuscript, we now report performance of our models on Clinvar SVs as suggested by the reviewer.

Generally, the manuscript misses in depth information about properties of model training. Besides the mentioned length distributions or unclear information about applied transformations in a prediction setting, very basic numbers like the number of training examples, information about data balancing, or model hyperparameters are missing. Steps like the introduced 10bp binning and averaging come without any motivation (computational considerations?).

We apologize for missing details in our method section. We have updated our manuscript to provide further details relevant for model building steps.

Change to manuscript: *Along with length distribution disparity, differences in the number of SVs between the disease and benign cohorts could bias our models. To address this challenge of data imbalance, we randomly sub-selected SVs from the 1KG SV and other control datasets such that the number of SVs belonging to the benign/control dataset equaled the corresponding number of disease-associated SVs. We applied this data balancing approach to both our somatic and germline models.*

*…………………….*

*Overall, the number of deletions was significantly higher than duplications in our disease and corresponding benign datasets (supplement data table 7).*

*…………………….*

*We also performed hyper-parameter tuning to optimize different somatic and germline models. For hyper-parameter optimization, we systematically varied the maximum depth of each tree, the total number of trees in the forest, and the minimum number of leaves required to split an internal node. We applied training datasets for each disease/benign cohort to build a comprehensive set of models.*

*……………………..*

*Subsequently, we calculated the average of these values for each dataset over all 10 bp bins that overlap with a given SV. We applied this 10 bp bin approach to increase the efficiency of our computation. Furthermore, this method provides us flexibility such that one could utilize the maximum or minimum of these bins as a feature instead of using the mean value applied in the current study.*

The paper is well written but at times repetitive, i.e. restating already given information without going into additional detail.

We thank the reviewer for this helpful comment. We have updated the manuscript to address the issue of repetitiveness in the main text.

Considering that the authors implicate pathogenic, or cancer SVs in this study it would be relevant to give access to these results (aggregated, i.e. without releasing an association with patients identifiers).

We thank the reviewer for this suggestion. We now provide a list of putative pathogenic SVs from the ClinVar dataset. We note that whole-genome cancer variants (from TCGA) are considered protected (regardless of patient identifier exclusion), and thus dissemination of their coordinate is very challenging.

Change to manuscript: *Finally, we also report a subset of highly pathogenic deletions and duplications in the ClinVar database (supplement data table8-9).*

**2^nd^ round**

**Reviewer 1**

The authors have made substantial improvements to the manuscript and have satisfactorily responded to concerns.

**Reviewer 2**

In this revision of the manuscript “SVFX: a machine-learning framework to quantify the pathogenicity of structural variants” Kumar and colleagues build a model to predict pathogenicity of structural variants on somatic and germline SVs from disease vs. healthy cohorts. The authors have addressed multiple issues raised by two reviewers. While I see improvements with this revision, I am not satisfied by the authors responses and edits.

1) The manuscript still misses relevant information. For example, the authors were supposed to add information about model training and hyperparameter optimization and edited the following sentences: "We also performed hyper-parameter tuning to optimize different somatic and germline models. For hyperparameter optimization, we systematically varied the maximum depth of each tree, the total number of trees in the forest, and the minimum number of leaves required to split an internal node." There is no information about the exact value ranges explored, no information about the selected hyperparameters or on which data the optimization was performed. This is not a valid description of methods. The authors have to make sure that someone reading the manuscript understands the rationale behind statistical and algorithmic decisions of the authors and is able to rebuild the proposed approach from these descriptions.

2) In the previous round of reviews, I asked to add information about training data set sizes and generally numbers of observations. This information is still generally missing from the main text. This is no information that should be hidden in supplemental tables.

3) The authors decided to add the ClinVar data as a validation data set. I had hoped they would do more than just that, but I am happy to see at least one more data set. Their results seem intriguing and while they were not able to provide their SV scores for the previous data sets, they decided to not provide all scores for the ClinVar data either. This makes it impossible to check the author's results or to compare to other methods. The complete scoring of this validation data set has to be made available.

4) While I have not tested its functionality, the referenced GitHub repository only provides code to annotate, i.e. produce a feature matrix, and to train a model. It does not provide the trained models or means to score custom SVs. While one of the major claims of this paper is "In particular, we generated somatic and germline training models, which included genomic, epigenomic, and conservation-based features for SV call sets in diseased and healthy individuals." –authors are not making these models (incl. required scaling) or a scoring routine available. I do not believe that to be appropriate.

5) I find the results regarding the effects of SV length to be contradicting: "[…] we quantified the predictability differences for the original models when the SV length was removed as a feature. Overall, we found that the AUC value was 3% lower, on average, for truncated somatic models that lacked SV length […] We observed that SV length and overlap with ultraconserved regions were the most significant contributors to the predictive performance of the model (supplement Fig S5)." How can one of the two most significant features only contribute 3% of the AUC performance?

**Authors’ response**

Reviewer 2:

The manuscript still misses relevant information. For example, the authors were supposed to add information about model training and hyperparameter optimization and edited the following sentences: "We also performed hyperparameter tuning to optimize different somatic and germline models. For hyperparameter optimization, we systematically varied the maximum depth of each tree, the total number of trees in the forest, and the minimum number of leaves required to split an internal node." There is no information about the exact value ranges explored, no information about the selected hyperparameters or on which data the optimization was performed. This is not a valid description of methods. The authors have to make sure that someone reading the manuscript understands the rationale behind statistical and algorithmic decisions of the authors and is able to rebuild the proposed approach from these descriptions.

We thank the reviewer for this suggestion. In the revised manuscript, we provide value ranges for various parameters that were explored during hyper parameter tuning step.

Change to manuscript: *For hyper-parameter optimization, we systematically varied the maximum depth of each tree (value ranges between 2 to 10), the total number of trees in the forest (in the range of 10 to 5000), and the minimum amount of leaves required to split an internal node ( in between 10 to 100). The primary considerations behind our choices of these hyperparameters were interpretability, tractability, and model performance. Maximum depth and the number of trees in a forest are intuitive, necessary, and well-documented controls of model complexity in tree-based and forest-based models. In particular, tuning these two parameters helps navigate the bias-variance tradeoff for an ensemblebased tree model. Similarly, varying the minimum number of leaves required to split an internal node influence the model performance.*

In the previous round of reviews, I asked to add information about training data set sizes and generally numbers of observations. This information is still generally missing from the main text. This is no information that should be hidden in supplemental tables.

We thank the reviewer for the feedback. We now explicitly report these numbers in the updated main text.

Change to manuscript: *For instance, our somatic dataset included 7295 deletions on average (maximum and minimum frequency of 11917and 4514 in BRCA and STAD cohorts, respectively) across all six cancer cohorts. In contrast, there were 5204 duplications on average (with maximum and minimum of 6023 and 3675 for BRCA and SKCM cohorts, respectively). Similarly, our germline dataset included on average 9094 deletions across multiple cancer types and 9306 deletions in the CVD cohort.*

The authors decided to add the ClinVar data as a validation data set. I had hoped they would do more than just that, but I am happy to see at least one more data set. Their results seem intriguing and while they were not able to provide their SV scores for the previous data sets, they decided to not provide all scores for the ClinVar data either. This makes it impossible to check the author's results or to compare to other methods. The complete scoring of this validation data set has to be made available.

We thank the reviewer for the feedback. However, we note that in the previous revision of this manuscript, we performed predictions on SVs from the inflammatory bowel disease cohort and the ClinVar dataset. Based on this reviewer’s suggestion, we now report SV score for the entire ClinVar SV database rather than only high scoring ones.

Change to manuscript: *Finally, we also report pathogenicity scores for deletions and duplications in the ClinVar database (supplement data tables 8 & 9).*

While I have not tested its functionality, the referenced GitHub repository only provides code to annotate, i.e. produce a feature matrix, and to train a model. It does not provide the trained models or means to score custom SVs. While one of the major claims of this paper is "In particular, we generated somatic and germline training models, which included genomic, epigenomic, and conservation-based features for SV call sets in diseased and healthy individuals." –authors are not making these models (incl. required scaling) or a scoring routine available. I do not believe that to be appropriate.

We apologize for the confusion. Previously, we were slightly hesitant to release the pretrained model due to privacy issue related to accidental dissemination of cancer SV coordinates. Currently, large scale TCGA whole genome data in form of small or large variants are prohibited from distribution. However, based on this reviewer’s feedback, we confirmed that SV coordinates can’t be extracted from pretrained model. Thus, we now provide various pretrained models through the SVFX github link. We highlight this point in the updated manuscript.

Change to manuscript: *Finally, we also provide pre-trained somatic and germline models through the SVFX Github page. We note that these pre-trained models disallow the extraction of training SV coordinates to avoid any potential variant leakage.*

I find the results regarding the effects of SV length to be contradicting: "[…] we quantified the predictability differences for the original models when the SV length was removed as a feature. Overall, we found that the AUC value was 3% lower, on average, for truncated somatic models that lacked SV length […] We observed that SV length and overlap with ultraconserved regions were the most significant contributors to the predictive performance of the model (supplement Fig S5)." How can one of the two most significant features only contribute 3% of the AUC performance?

We thank the reviewer and apologize for the confusion. We understand the reviewer's concern. However, we disagree with the reviewer's assertion that our results are contradictory. In the previous round of review, this reviewer raised concern regarding ascertainment bias in our model due to length mismatch between cancer 1KG dataset. To address this concern, we removed length as an explicit to feature and built the truncated model and observed a 3% decrease in AUC. We interpreted this result as the robustness of our model, where scoring is not just dependent on length as a feature. However, the reviewer understood that "the two most significant features only contribute 3% of the AUC performance."

First, in our length-truncated model, the conservation-related feature remains, which, to a certain extent, avoids a drastic change in the AUC value. Second, our observation from the length-truncated model does not indicate that "most significant features only contribute 3% of the AUC performance" because there can be redundancy in the features, meaning that SV length may be correlated with (or mutually dependent) other features.

One simple and obvious example of this is the collinearity, where one feature can be explained as a linear combination of other feature(s). To evaluate the dependency among features, we computed the spearman feature correlation matrix. We observed that SV length correlates with various features, including coding regions (r = 0.13), splice sites (r = 0.12), promoters (r = 0.11), and PhyloP conservation scores (r = 0.17). Furthermore, these correlated features are themselves are in correlation with regulatory and functional genomics signals. Note that feature importance, as we have compiled it, is the mean decrease in entropy caused by the feature among nodes in all trees in the forest, weighted by the fraction of data points that "reach" those nodes. It is thus important to note that a strongly correlated feature will have strong feature importance at the expense of another feature’s importance.

Additionally, we also observed that a simple multivariate linear model that predicts SV length captures 20-25% of the variation in the SV length. Thus, even a simple linear combination of other features can explain a substantial difference in SV length. These results suggest that the SV length's removal does not mean the effect of SV length is removed from the SV effect prediction because SV length is encoded in other features. Thus, a 3% AUC decrease does not reflect the absolute contribution of SV length on the impact estimation. It instead reflects the accuracy contributed exclusively by the SV length and not by any other feature. In this sense, a small 3% drop in AUC indicates that our model does not only use SV length as the dominating feature. On the contrary, other features also carry important information about the functional impact of the SVs.

Finally, the analysis of the feature importance and selection in random forests are complex problems that are extremely hard to analyze (Genuer et al.). While we agree that a systematic understanding of the feature importance is a critical issue in machine learning, we believe this discussion is out of the scope of our study.

Reference

Robin Genuer, Jean-Michel Poggi, Christine Tuleau-Malot. Variable selection using Random Forests. Pattern Recognition Letters, Elsevier, 2010, 31 (14), pp.2225-2236.

**3^rd^ round**

**Reviewer 2**

In this second revision of the manuscript “SVFX: a machine-learning framework to quantify the pathogenicity of structural variants” Kumar and colleagues present multiple random forest models to predict pathogenicity of structural variants on somatic and germline SVs from disease vs. healthy cohorts. The authors have again addressed my comments; however, in what I feel was rather dismissive letter of response and letter to the editor.

Despite all that, I highly appreciate that the authors have released their trained models, improved descriptions in their GitHub repository, and made scores for the complete ClinVar data set available as a Supplement.

I hope that the authors give their methods another read through and remind themselves that our profession depends on principles of open and reproducible research. A long these lines, I will point out again that the authors still fail to describe the parameters that were chosen from their hyperparameter search. Handling of missing values/imputation, e.g. as a separate "missing state" as possible with tree models is not mentioned. They also do not describe software/packages used.

While I think good method descriptions are the most important point, I want to clarify briefly the point that I wanted to make about the "contradiction" with ~15% feature importance for length and saying that removing length as an explicit feature only reduced the AUC by 3%. The authors claim at multiple occasions that they handled a potential length bias in their training data set: e.g. "to avoid the effect of such length bias on our models", "to avoid any implicit feature bias due to length distribution", "Z-score-normalized feature matrices helped us avoid implicit bias". Then they see length as one of their most important features and notice that removing it must be compensated by other correlated features (without actually saying or showing that in the manuscript text and only in the response to me). I have to say that I am not convinced that the remaining length effect is purely biological/real. For that matter, the effects of Z-score transformation on non-normal distributed features and effects of length-correlated features remain underexplored by the authors.
